# A Wave–Particle Model of Energy Transfer Between Two Atoms in a Transactional Interpretation of Quantum Mechanics

**DOI:** 10.3390/e28070813

**Published:** 2026-07-17

**Authors:** Lloyd Watts, Carver Mead

**Affiliations:** 1 Neocortix, Inc., 800 W. El Camino Real Suite 180, Mountain View, CA 94040, USA; lwatts@neocortix.com; 2 California Institute of Technology, Pasadena, CA 91125, USA

**Keywords:** photon, structure, quantum, transition, electromagnetic, wave, atom, emitter, absorber

## Abstract

In 2000, Carver Mead introduced a time-symmetrical theory of energy exchange between two atoms, building on the *Transactional Interpretation of Quantum Mechanics* by John Cramer in 1986. In 2020, Cramer and Mead developed the theory further, proposing a conceptual path integral formulation by which energy could be completely transferred over long distances, and showing that this theory can explain the Einstein–Podolsky–Rosen paradox, the Hanbury-Brown–Twiss effect, and the Freedman–Clauser entanglement experiment. In this paper, we develop the theory further, proposing a specific formulation of the interaction between Emitter and Absorber Atoms, in which the energy density is proportional to the root-mean-square of the product of retarded and advanced four-vector potential waves, and show how this interaction efficiently and completely transfers energy from the Emitter Atom to the Absorber Atom over arbitrary distances. We use Mach’s Principle and conservation of energy to find the proportionality constant by matching the mean transition time constant for all possible Absorbers in the universe to the mean transition lifetime computed from Fermi’s Golden Rule, leading to a complete solution with no adjustable parameters. The solution represents the exchange of energy between two atoms, valid over 26 orders of magnitude in Emitter–Absorber distance, from about 0.52 m to the radius of the Hubble Sphere $1.27\times 10^{26}$ m. We define this Wave–Particle Model as the product of a retarded Emitter vector potential wave and an advanced Absorber vector potential wave, which exhibits the particle-like properties of losslessly carrying energy at the speed of light in a straight line from Emitter Atom to Absorber Atom in a vacuum in the absence of gravity.

## 1. Introduction

In 2000, Carver Mead introduced a time-symmetrical theory of energy exchange between two atoms [1], building on the *Transactional Interpretation of Quantum Mechanics* by John Cramer in 1986 [2]. In 2020, Cramer and Mead developed the theory further [3], proposing a conceptual path integral formulation by which energy could be completely transferred over long distances, and showing that this theory can explain the Einstein–Podolsky–Rosen paradox [4], the Hanbury-Brown–Twiss effect [5], and the Freedman–Clauser entanglement experiment [6]. In this paper, we develop the theory further, proposing a specific formulation of the interaction between Emitter and Absorber Atoms, in which the energy density is proportional to the root-mean-square of the product of retarded and advanced four-vector potential waves, and show how this interaction efficiently and completely transfers energy from the Emitter Atom to the Absorber Atom over arbitrary distances.

Cramer and Mead 2020 [3] give a thorough description of the unsolved problems with Quantum Mechanics, the motivation and justification for the Transactional Interpretation of Quantum Mechanics (TIQM), and the suitability of the two-atom energy transfer problem to illustrate that a combination of retarded Emitter waves and advanced Absorber waves can provide a mechanism for wavefunction collapse. Cramer and Mead 2020 [3] proposed that the energy in transit between the two atoms would be proportional to the *sum of the retarded Emitter wave and the advanced Absorber wave*. Unfortunately, this solution did not conserve energy and it was not valid for large Emitter–Absorber distances.

In this paper, we propose that the energy in transit between the two atoms would be proportional to the *root-mean-square of the product of the retarded Emitter wave and the advanced Absorber wave*, and we show that this solution does conserve energy and is valid over 26 orders of magnitude in Emitter–Absorber distance, from about 0.52 m to the radius of the Hubble Sphere $1.27\times 10^{26}$ m. Following Cramer and Mead 2020 [3],

*We show that this approach can provide a detailed mathematical description of a “quantum-jump” in which what seems to be a photon is emitted by one hydrogen atom in an excited state and excites another hydrogen atom initially in its ground state. Thus, the mysterious process of wave function collapse becomes just a phenomenon involving an exchange of advanced/retarded electromagnetic waves between atomic wave functions described by the Schrödinger formalism.*

We must acknowledge that the problem of energy transfer between two atoms is regarded by the modern physics community as solved long ago by Quantum Electrodynamics (QED). Quantum Electrodynamics is the theory of the interaction of light and matter, broadly accepted by the modern physics community since its introduction in the late 1940s (Feynman 1949 [7], Feynman 1950 [8]). In QED, atomic quantum transitions are instantaneous events that emit and absorb photons, which are structureless elementary particles that carry discrete quanta of energy at the speed of light away from an Emitter Atom, where they may or may not be absorbed by an Absorber Atom. QED provides very accurate predictions of energy levels and photon emission and detection probabilities (Feynman 1985 [9], Milonni 1993 [10]). Modern extensions of QED have shown that transmission of photons is causal, i.e., photons cannot be detected at an Absorber Atom sooner than the transit time at the speed of light (Power and Thirunamachandran 1997 [11]). And a photon conveys light energy and its path is affected by gravity, as described by Einstein’s General Theory of Relativity.

But for all of its great successes, QED has known problems and limitations, some of which are addressed by our present work:

1. **QED is not self-consistent.** QED assumes a point-particle model of an electron, and then develops a solution that contradicts that assumption. In QED, the Lamb Shift is understood as based on electron self-interaction, in which the electron “continuously emits and absorbs virtual photons, and as a result its electric charge is spread over a finite volume instead of being pointlike” (Eides, Grotch, and Shelyuto, 2007, [12]). From Milonni 1993 [10], “Welton (1948) interpreted the Lamb Shift as follows. The vacuum field causes the position of the electron to fluctuate. … Now the fluctuation in r causes the potential energy V(r) to fluctuate…”. QED does not compute a corrected non-singular wavefunction that accounts for this non-pointlike charge distribution necessary to explain the Lamb Shift (Watts, 2016, [13]).
2. **Renormalization is not mathematically self-consistent.** Dirac objected strongly to Renormalization, saying in 1978 [14]:

*I must say that I am very dissatisfied with the situation, because this so-called ‘good theory’ does involve neglecting infinities which appear in its equations, neglecting them in an arbitrary way. This is just not sensible mathematics. Sensible mathematics involves neglecting a quantity when it is small—not neglecting it just because it is infinitely great and you do not want it!*

Even Feynman said in 1985 [9]:

*The shell game that we play … is technically called ‘renormalization’. But no matter how clever the word, it is still what I would call a dippy process! Having to resort to such hocus-pocus has prevented us from proving that the theory of quantum electrodynamics is mathematically self-consistent. It’s surprising that the theory still hasn’t been proved self-consistent one way or the other by now; I suspect that renormalization is not mathematically legitimate. What is certain is that we do not have a good mathematical way to describe the theory of quantum electrodynamics: such a bunch of words to describe the connection between n and j and m and e is not good mathematics.*

3. **Quantum mechanical objects are given a misleading name, “particles”, which leads to real confusion in the field, and makes it easy to prematurely dismiss legitimate attempts to model their known wavelike behavior.** From Feynman, 1985 [9]:

*It’s rather interesting to note that electrons looked like particles at first, and their wavish character was later discovered. On the other hand, apart from Newton making a mistake and thinking that light was “corpuscular,” light looked like waves at first, and its characteristics as a particle were discovered later. In fact, both objects behave somewhat like waves, and somewhat like particles. In order to save ourselves from inventing new words such as “wavicles,” we have chosen to call these objects “particles,” but we all know that they obey these rules for drawing and combining arrows that I have been explaining. It appears that all the “particles” in Nature—quarks, gluons, neutrinos, and so forth—behave in this quantum mechanical way.*

By this logic, a quantum mechanical object such as a photon or an electron is neither a classical particle nor a classical wave; it is something else that has both particle-like and wave-like properties. For something so important, so confusing, and so different from everyday experience, it would have been a very good idea to give it a new name.

4. **QM/QED shows how to accurately calculate atomic energy levels and probabilities of detecting photons at different places and times, but it does not give:**
  **a.**  **A corrected wavefunction model of an electron in a Hydrogen Atom, consistent with fine structure, spin 1/2 and Lamb Shift.**
  **b.**  **A mathematical description of how an atom emits a photon.**
  **c.**  **A mathematical description of how an atom absorbs a photon.**
  **d.**  **A mathematical description of how a photon travels from Emitter Atom to Absorber Atom carrying a frequency-dependent energy E = h ν, consistent with single- and double-slit experiments.**

From Feynman 1985 [9]:

*The more you see how strangely Nature behaves, the harder it is to make a model that explains how even the simplest phenomena actually work. So theoretical physics has given up on that.*

QED is not a mathematical/physical model of electrons and photons; it is a way of calculating electron and photon energy levels and probabilities.

5. **QED cannot explain the Einstein–Podolsky–Rosen paradox, the Hanbury-Brown–Twiss effect, and the Freedman–Clauser entanglement experiment.**
6. **The Cosmological Constant Problem is unresolved:** from Milonni 1993 [10]:

*The reality of zero-point energies suggested by the existence of Casimir forces evidently means that zero-point energies should be taken seriously in general relativity. When this is done the total zero-point energy density of the vacuum acts in effect as a cosmological constant of the type introduced by Einstein in order to have static solutions of his field equations. However, astronomical data indicate that any such cosmological constant must be many orders of magnitude smaller than predicted by quantum field theory (Weinberg, 1989). This difficulty remains unresolved.*

7. **Standard QED cannot overcome Haag’s Inconsistency Theorem:** Kastner 2015 [15] provides a critique of standard QED and a demonstration that inconsistencies related to Haag’s theorem and Fock spaces are resolved in TIQM. It also notes that non-local direct-action theories such as TIQM are immune to the self-energy problem of standard gauge field QED, and can also provide a solution to the problem of gauge arbitrariness.

So, Quantum Mechanics and Quantum Electrodynamics allow us to calculate with great accuracy the energy levels and magnetic moments in the Hydrogen Atom, but the theory is not mathematically self-consistent, it cannot explain many modern experiments, and it does not attempt to give a mathematical/physical model of the electron and photon consistent with single- and double-slit experiments that could be understood or visualized, even by its creators. As far as we know, these problems are still unsolved, even in 2026. This is the motivation and justification for our broader research.

And specifically, our current paper addresses points 4(b), 4(c), and 4(d) above, repeated here:

**4(b):**  **A mathematical description of how an atom emits a photon.**
**4(c):**  **A mathematical description of how an atom absorbs a photon.**
**4(d):**  **A mathematical description of how a photon travels from Emitter Atom to Absorber Atom carrying a frequency-dependent energy E = h υ, consistent with single- and double-slit experiments.**

Note that our present work is not contradictory to the successful results of QED. QED is a remarkably successful theory, which makes very accurate predictions that agree very well with experiment. Our present work agrees with the successful results of QED, while providing a deeper mathematical model of photon emission, transmission, and absorption, which goes beyond the limitations of QED, and provides an explicit mathematical/physical model of wave–particle duality, which is not provided by QED. Our present work builds on the causal, non-local framework of Transactional Interpretation of Quantum Mechanics to achieve the reconciliation of particle-like and wave-like behavior of photons, which is not done in QED. We note that Kastner 2012 [16] claims that by considering the product of the advanced and retarded wavefunctions, the Born rule can be explained ontologically, thus anticipating our approach of defining the energy as the root-mean-square of the product of the retarded Emitter vector potential wave and the advanced Absorber vector potential wave. Kastner 2022 [17] further showed that energy comes from the product of the Offer Wave and Confirmation Wave, based on an earlier analysis in Kastner and Cramer 2018 [18].

By defining a photon as a structureless elementary particle, the founders of QED created the very situation that it is impossible to build a mathematical model of photon transmission that exhibits the wave-like behavior seen in single- and double-slit experiments. To escape this deadlock, we start with a different premise. We begin with a wavefunction model of two Hydrogen Atoms, and build a new mathematical/physical model of the energy transfer, which has the wave-like behavior built-in from the start, such that it behaves like a particle in the simplest case of transmission through a vacuum in the absence of gravity, namely that it travels in a straight line from Emitter to Absorber at the speed of light, carries all of the Emitter transition energy from Emitter to Absorber, and deposits the energy into the Absorber Atom. Note that this is not a semi-classical approach, or a simple revisitation of 1920s physics. This is a fundamentally new approach, which combines retarded and advanced vector potential waves in a new way to create a new kind of non-classical directed energy-carrying wave with remarkable particle-like properties, hence worthy of a new name, which we will call a Wave–Particle Model. Recall that Feynman said in 1985 [9] that “theoretical physics has given up on [making] a model that explains how even the simplest phenomena actually work.” Our new mathematical/physical Wave–Particle Model of the wave-like and particle-like transmission of energy between two atoms is a clear example of the very endeavor that Feynman said theoretical physics had given up on.

Finally, we note the limited scope of our current paper. We are not addressing all of the known problems and limitations of QED in this paper, and, therefore, we are not suggesting that this paper alone would justify the replacement of QED with our transactional theory. Our current work is an important early step toward a “future, more refined theory” (Milonni, 1993 [10]), which would address all of the known problems and limitations of QED.

## 2. Vector Potential Waves and Their Interactions

**Figure 1:**
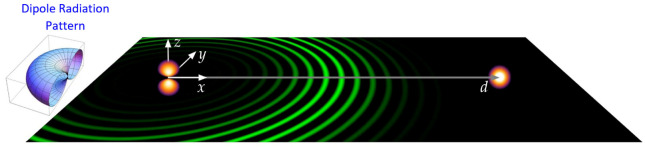
Coordinate system: The excited Emitter Atom is located at the coordinate system origin, and when in a superposition state with the ground state, it creates an oscillating electric dipole oriented along the *z* axis, which has a maximum radiation amplitude in the *x*–*y* plane, and no transmission in the *z* direction. The Absorber Atom is located a distance *d* away from the origin along the positive *x* axis.

**Figure 2:**
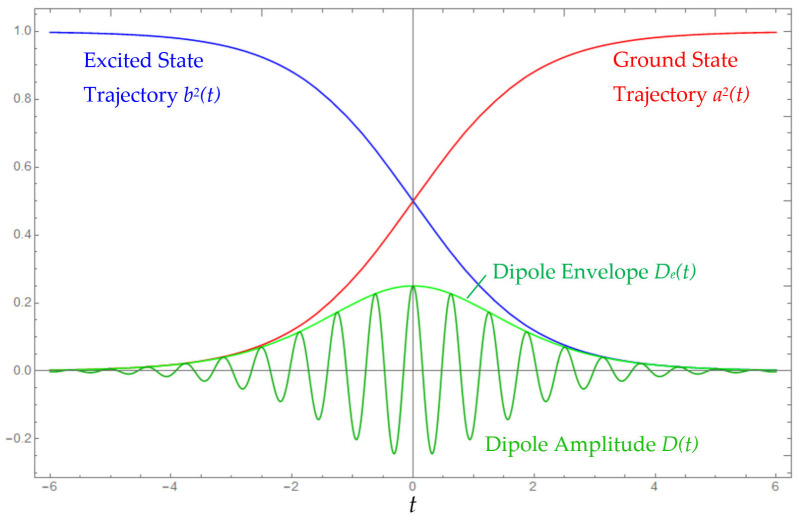
The time trajectory of the Emitter Atom as it undergoes an atomic transition from excited state <210> to ground state <100>. The Dipole Amplitude pulse drives the outgoing four-vector potential wave.

In Figure 1, we establish the coordinate system and general framework for the analysis. The excited Emitter Atom is located at the coordinate system origin. It is shown in the excited <210> state, in the conventional orientation aligned with the *z* axis. This state is stationary, with no oscillating dipole motion. When the Emitter Atom is perturbed, such that it enters a superposition state with the ground state <100>, an oscillating electric dipole is formed, whose motion is also oriented along the *z* axis. This causes an outward radiation wave of the four-vector potential, which has the well-known Hertzian Dipole Radiation pattern, which has its maximum radiation strength in the *x*–*y* plane, with no radiation in the *z* direction. For this reason, we will draw slices of the propagating four-vector potential waves in the *x*–*y* plane. The green rings in Figure 1 represent the outward-propagating four-vector potential wave that would result from a short pulse of dipole activity, as would occur when the Emitter Atom transitions from the excited <210> state to the ground state <100>, as described in Figure 2.

In Figure 2, we show the relationship between the evolving excited and ground states of the Emitter Atom and the Dipole Envelope and waveform amplitude, as a function of time. At the beginning of the transition, the excited state (blue) dominates with an amplitude nearly = 1, and the ground state (red) has an amplitude of nearly = 0. The Dipole Envelope (light green), proportional to the product of the blue and red curves, is nearly zero at the beginning of the transition. As the transition progresses, the excited state loses energy, and the ground state gains energy, as the Dipole Envelope grows to its peak envelope size at t = 0. The Dipole Amplitude (dark green) vibrates at the difference frequency between the excited and ground states, and reaches a maximum amplitude at t = 0. After t = 0, the transition continues, as the excited state loses all of its energy, the ground state becomes fully developed, and the dipole vibrations die out. The Emitter Atom is now in the ground state and is no longer vibrating as an electric dipole.

We will now describe the Emitter Atom transition mathematically. From [3], we assume a form derived for constant coupling of the vector potential all the way from Emitter to Absorber. Once we have solutions for the advanced and retarded fields and their interaction, we can check this assumption. From [3], the excited state trajectory $b^{2}\left(t\right)$ and ground state trajectory $a^{2}\left(t\right)$ are given by

(1) $$b^{2}\left(t\right)=\frac{1}{\left(e^{t/\tau }+1\right)}a^{2}\left(t\right)=\frac{1}{\left(e^{-t/\tau }+1\right)}$$

where τ is the time constant of the transition, and sets the overall duration of the photon pulse. τ will be determined later by conservation of energy considerations. The Dipole Envelope $D_{e}\left(t\right)$ is given by

(2) $$D_{e}\left(t\right)={D_{0}a}^{2}b^{2}=\frac{D_{0}}{\left(e^{t/\tau }+1\right)\left(e^{-t/\tau }+1\right)}$$

where $D_{0}$ is a scaling constant, which sets the peak amplitude of the dipole vibrations. The Dipole Amplitude D(t) is given by

(3) $$D\left(t\right)=D_{e}cos\left(\omega t\right)=\frac{D_{0}\;cos\left(\omega t\right)}{\left(e^{t/\tau }+1\right)\left(e^{-t/\tau }+1\right)}$$

where ω is the difference frequency between the excited <210> and ground <100> states. We will be using the Dipole Amplitude D(t) to drive the outgoing four-vector potential wave from the Emitter Atom. In practice, ω≫1/τ, i.e., there are a great many cycles of Dipole Amplitude wave inside the Dipole Envelope pulse, but for illustrative clarity, we will draw the figures with a much smaller number of waveform cycles in the pulse.

### 2.1. The Outgoing Emitter Vector Potential Wave

Now we want to describe the Emitter’s outgoing four-vector potential wave, as driven by the Dipole Amplitude D(t) at the origin. This is essentially a boundary value problem with the relativistically correct Reimann–Sommerfeld second-order differential equation governing all of space:

(4) $$\left(\nabla^{2}-\frac{\partial^{2}}{{\partial t}^{2}}\right)A=-\mu_{0}\;J$$

where $A=\left[\vec{A},V/c\right]$ is the four-potential and $J=\left[\vec{J},c\rho \right]$ is the four-current, $\vec{A}$ is the vector potential, V is the scalar potential, $\vec{J}$ is the physical current density (no displacement current), and ρ is the physical charge density, all expressed in the same inertial frame.

The general Green’s Function solution for the four-potential A(t) at a point in space from the four-current density J(r,t) in volume elements, dvol at a distance r from that point is

(5) $$A\left(t\right)=\frac{\mu_{0}}{4\pi }\int \frac{J\left(r,t\pm r/c\right)}{r}dvol$$

where r is the distance from element dvol to the point where A is evaluated, assumed large compared to the size of the atomic wave functions, and c is the speed of light.

Equation (5) is the first fundamental equation of electromagnetic coupling: The vector potential, which will appear as part of an electron’s momentum, is simply the sum of all current elements on that electron’s light cone, each weighted inversely with its distance from that electron. The second-order nature of derivatives in Equation (4) does not favor any particular sign of space or time. Thus, the four-potential from a current element on the past light cone of the electron (t−r/c) will be “felt” by the electron at later time t, and is termed a **retarded** field. Conversely, the four-potential from a current element on the future light cone of the electron (t+r/c) will be “felt” by the electron at earlier time t, and is termed an **advanced** field. Historically, with rare exception, advanced fields have been discarded as non-physical because evidence for them has been explained in other ways. We shall see that modern quantum experiments provide overwhelming evidence for their active role in **quantum entanglement**.

The Green’s Function integral forms for the retarded scalar and vector potentials are

(6) $$V\left(t\right)=\frac{1}{4\pi \epsilon_{0}}\int \frac{\rho \left(t-r/c\right)}{r}dvol\;\;\;\;\;\;\;\;\;\;\;\;\;\;\;\;\;\;\vec{A}\left(t\right)=\frac{\mu_{0}}{4\pi }\int \frac{\vec{J}\left(t-r/c\right)}{r}dvol$$

We consider a compact charge distribution that is changing with time, and inquire about the general properties of the far-field radiation from that charge distribution. By far-field, we mean that the distance r from any point in the source to the point of observation is larger than any dimension of the source by a large enough factor that, for any two points in the source, 1/r is constant to within the accuracy required. We choose our coordinate system so that the vector from the source to the point of observation is in the x direction. We divide the source into thin slices with planes of constant x, such that each slice of thickness x contains charge q(x,t). We choose the origin x=0 such that its distance to the point of measurement is R. From Equation (6), the potential V contributed by the charge q(x,t) in a single slice located at x at time t will be delayed by the propagation time (R−x)/c:

(7) $$V\left(t\right)=\frac{1}{4\pi \epsilon_{0}R}q\left(t-\left(R-x\right)/c\right)=\frac{1}{4\pi \epsilon_{0}R}q\left(t-R/c+x/c\right)$$

Therefore, the potential $V_{1}$ contributed by the charge $q_{1}\left(x,t\right)$ in a single slice located at x is

(8) $$V_{1}\left(t^{\prime }\right)=\frac{1}{4\pi \epsilon_{0}R}q_{1}\left(t\right)$$

where $t=t^{\prime }-R/c+x/c$. The potential at the same observation point from the immediately adjacent slice (2) located at x+δx is

(9) $$V_{2}\left(t^{\prime }\right)=\frac{1}{4\pi \epsilon_{0}R}q_{2}\left(t+\delta x/c\right)$$

The total potential V at the point R will be

$${V=V_{1}+V}_{2}=\frac{1}{4\pi \epsilon_{0}R}\left(q_{1}\left(t\right)+q_{2}\left(t+\delta x/c\right)\right)$$

(10) $$\;=\frac{1}{4\pi \epsilon_{0}R}\left(q_{1}\left(t\right)+q_{2}\left(t\right)+\frac{\delta x}{c}\frac{\partial q_{2}}{\partial t}\right)$$

$$\frac{\partial V}{\partial t^{\prime }}=\frac{1}{4\pi \epsilon_{0}R}\left(\frac{\partial q_{1}}{\partial t}+\frac{\partial q_{2}}{\partial t}+\frac{\delta x}{c}\frac{\partial^{2}q_{2}}{\partial t^{2}}\right)$$

If we consider these two adjacent slices in isolation, the charge on the first will be decreased, and that on the second will be increased by a current I crossing the boundary between them in the +x direction.

By conservation of charge

(11) $$\frac{\partial q_{2}}{\partial t}=-\frac{\partial q_{1}}{\partial t}=I$$

which, with Equation (12), gives

(12) $$\frac{\partial V}{\partial t^{\prime }}=\frac{\delta x}{4\pi \epsilon_{0}Rc}\frac{\partial^{2}q_{2}}{\partial t^{2}}$$

The potential V is propagating in the +x direction at velocity c. Its functional form is therefore V=f(t−R/c). The derivatives are thus related by

(13) $$\frac{\partial V}{\partial R}=-\frac{1}{c}\frac{\partial V}{\partial t^{\prime }}=-\frac{\delta x}{4\pi \epsilon_{0}c^{2}R}\frac{\partial^{2}q_{2}}{\partial t^{2}}$$

where we have used the fact that $\mu_{0}\epsilon_{0}=1/c^{2}$.

By Equations (6) and (11), the vector potential at point R will be

(14) $$\vec{A}\left(R,t^{\prime }\right)=\frac{\mu_{0}}{4\pi R}I\left(t\right)\delta x=\frac{\mu_{0}\delta x}{4\pi R}\frac{\partial q_{2}}{\partial t}$$

(15) $$\frac{\partial \vec{A}}{\partial t}=\frac{\mu_{0}\delta x}{4\pi R}\frac{\partial^{2}q_{2}}{\partial t^{2}}$$

From Equations (13) and (15), we conclude that the total electric field $\vec{E}$ in the direction of propagation vanishes:

(16) $$\frac{\partial \vec{A}}{\partial t}=-\frac{\partial V}{\partial R}\;\;\;\;\;\;\;\;\;\;\Rightarrow \;\;\;\;\;\;\;\;\vec{E}=-\nabla V-\frac{\partial \vec{A}}{\partial t}=0$$

We have thus arrived at a very important result for an electromagnetic wave propagating in free space far from its source:

**Transverse Theorem:** The electromagnetic wave propagating in free space due to a compact source has the following property:

*The time derivative of the longitudinal vector potential just cancels the spatial gradient of the scalar potential; therefore, the electric field in the direction of propagation is zero.*

The far-field radiation from any source approaches a plane wave arbitrarily closely at large distances from its source. The scalar potential V in a plane wave is constant within any plane normal to the direction of propagation. The only attribute of the wave that can affect charges is the transverse vector potential $A_{⊥}$, and the only non-zero component of the electric field is $E_{⊥}=-{\partial A}_{⊥}/\partial t$. Plane electromagnetic waves are thus said to be Transverse Waves. From Green’s Function (Equation (5)), it follows that we can find the components of the vector potential that interact with matter by considering only the components of the source current normal to the direction of propagation. This deep result enables an enormous simplification in calculating any far-field radiation pattern. The special case of this result for an electric dipole source is shown in [1].

This conclusion is with regard to propagating wave solutions. It obviously does not apply to the static electric field of a charge distribution with a net charge that is not varying with time.

For the Emitter Atom, the driving dipole is the current $\vec{I}$ moving along its length l:

(17) $$\vec{D}=\vec{I}l$$

We can express the transverse vector potential $A_{⊥}$ in terms of the dipole $\vec{D}$:

(18) $$A_{⊥}=A_{\theta }=\frac{\mu_{0}}{4\pi }\frac{\vec{D}\left(t\pm r/c\right)\cdot \hat{\theta }}{r}$$

where $A_{⊥}$ is in units of (Volt second)/meter, and the unit vector $\hat{\theta }$ is directed North–South on the surface of the sphere. We can now use the above electric dipole result to solve the boundary value problem in Equation (4). For a steady-state dipole oscillation with constant envelope:

(19) $$\vec{D}\left(r=0,t\right)=\vec{I}l=D_{0}\;cos\left(\omega t\right)\;\hat{z}$$

The solution is

(20) $$A_{⊥}\left(x,t\right)=-\frac{\mu_{0}\omega^{2}D_{0}}{4\pi r}cos\left(\omega t_{E}\right)\;\hat{z}\cdot \hat{\theta }$$

(21) $$t_{E}=t-\frac{r}{c}$$

where $t_{E}$ is the time coordinate relative to the departure of the pulse from the Emitter Atom. Note that the solution has the expected 1/r amplitude dependence.

Now, we can write the solution for our real problem, with the non-constant pulsed dipole time dependence at the origin. The modified input is

(22) $$\vec{D}\left(r=0,t\right)=\frac{D_{0}\;cos\left(\omega t\right)\;\hat{z}}{\left(e^{t/\tau }+1\right)\left(e^{-t/\tau }+1\right)}$$

**Figure 3:**
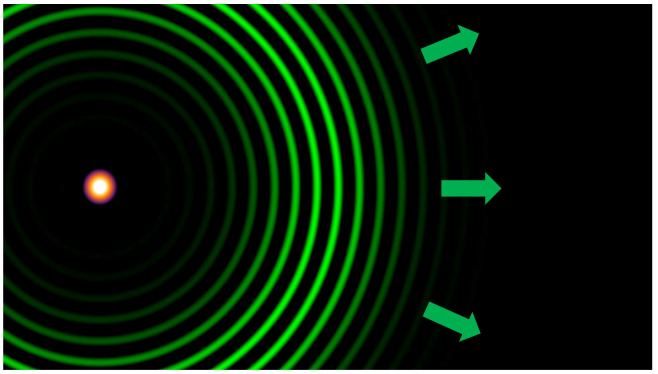
Vector potential retarded wave, generated by the current in the Emitter Atom, seen in the *x*–*y* plane, looking down the *z* axis from above. The wave propagates outward from the Emitter Atom at the speed of light, with a 1/r amplitude fall-off. This wave can be seen as an animation in [19].

And the corresponding solution $A_{{⊥}_{E}}\left(x,t\right)$ for the retarded Emitter wave is

(23) $$A_{{⊥}_{E}}\left(x,t\right)=-\frac{\mu_{0}\omega^{2}D_{0}\;cos\left(\omega t_{E}\right)\;\hat{z}\cdot \hat{\theta }}{4\pi r\;\left(e^{t_{E}/\tau }+1\right)\left(e^{-t_{E}/\tau }+1\right)}$$

i.e., we have an outward-propagating pulse from the origin, traveling at the speed of light, shaped in space like the temporal Dipole Amplitude pulse shape from Figure 2, with a 1/r amplitude fall-off, as shown in Figure 3. This solution is valid for the condition ω≫1/τ, i.e., the Dipole Envelope changes slowly compared to the dipole vibration period.

Historically, this outward-propagating vector potential retarded wave was assumed to carry the Emitter Atom’s transition energy away in all directions according to the dipole radiation pattern. In the new theory, this retarded wave from the Emitter Atom interacts with the inward-propagating vector potential advanced wave propagating into the Absorber Atom, such that it focuses the energy transfer from Emitter to Absorber.

Now we will describe the inward-propagating vector potential of the advanced wave propagating into the Absorber Atom.

### 2.2. The Incoming Absorber Vector Potential Wave

**Figure 4:**
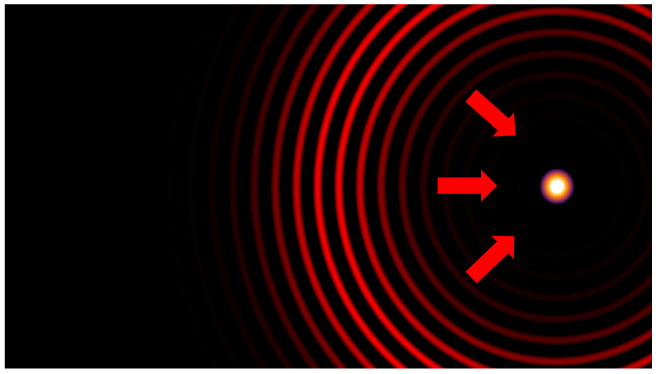
Vector potential advanced wave, generated by the current in the Absorber Atom, seen in the *x*–*y* plane, looking down the *z* axis from above. The wave propagates inward toward the Absorber Atom at the speed of light, growing in amplitude with a ${1/r}_{A}$ dependence. This wave can be seen as an animation in [19].

Figure 4 shows the inward-propagating vector potential advanced wave propagating into the Absorber Atom, as seen in the *x*–*y* plane, looking down the *z* axis from above. The wave propagates inward toward the Absorber Atom at the speed of light, growing in amplitude with a ${1/r}_{A}$ dependence, where $r_{A}$ is defined below.

We will place the Absorber Atom at position (x,y,z)=(d,0,0), i.e., at a distance d away from the Emitter Atom, on the positive *x* axis. Now, we can define a new radius $r_{A}$ relative to the position of the Absorber Atom:

(24) $$r_{A}=\sqrt{{\left(x-d\right)}^{2}+y^{2}+z^{2}}$$

(25) $$t_{A}=t+\frac{r_{A}}{c}$$

where $t_{A}$ is the time coordinate relative to the arrival of the pulse at the Absorber Atom. The corresponding solution $A_{{⊥}_{A}}\left(x,t\right)$ for the advanced Absorber wave is

(26) $$A_{{⊥}_{A}}\left(x,t\right)=-\frac{\mu_{0}\omega^{2}D_{0}cos\left(\omega t_{A}\right)\;\hat{z}\cdot {\hat{\theta }}_{A}}{4\pi r_{A}\;\left(e^{t_{A}/\tau }+1\right)\left(e^{-t_{A}/\tau }+1\right)}$$

where the angle ${\hat{\theta }}_{A}$ is defined relative to the shifted origin at the position of the Absorber.

### 2.3. The Interaction Between the Emitter and Absorber Waves

Mead and Cramer/Mead put forth a quantum-based electrodynamics formulated as strictly action-at-a-distance, mediated by the four-vector potential [3]. The prevalent formulation of QM also uses the vector potential, but resorts to the Poynting vector to provide an energy for electromagnetic modes of coupling [20]. This choice, from traditional Maxwell practice, is clearly a non-starter, as it gives a zero-point energy whose contribution to the cosmological constant is at least 60 orders of magnitude too large. This is currently a severe open problem in cosmology [21].

Using the vector potential avoids this problem because a potential has no degrees of freedom of its own—it is just a representation of the real four-current in each source, acting at a distance. It only creates an energy at positions where there is another four-current on its light-cone.

Working with strictly action-at-a-distance leaves a number of open questions of its own. When electromagnetic energy is transferred at the speed of light, where is it between when it leaves the source and when it arrives at its destination? We observe that its velocity and direction are influenced by the gravitational potential along its path—how can this occur if the energy is not present along the path? There are deep fundamental questions here that motivate us to formulate an energy-in-transit “field” view reminiscent of the Poynting vector, but symmetrical in time as expected from the symmetry of relativity [22,23].

In [3], Cramer and Mead proposed that the energy transfer is due to the sum of two terms: the retarded vector potential of the Emitter times the current in the Absorber, minus the advanced vector potential of the Absorber times the current in the Emitter. Because the vector potential is simply a representation of the current at a distance, the result is simply a product of the two currents with a coupling that is advanced/retarded and decreases inversely with separation.

An alternative view would be to attribute the energy to the product of the two vector potentials (suitably scaled), which would then flow from Emitter to Absorber along paths expected for a “photon particle.” Just as the completed transfer is given by the product of $A_{{⊥}_{E}}\cdot J_{{⊥}_{A}}$, the energy in transit is $\int A_{{⊥}_{E}}\cdot A_{{⊥}_{A}}$. This makes the entire transfer a continuous process, all the way from Emitter Atom to Absorber Atom. The present paper is an exploration of this approach.

From [3], Equation (51), the power transferred by the vector potential in dipole 2 to the current in dipole 1:

(27) $${\;\;\;\;\;\;\;\;\;\;\;\;\;\;\;P}_{1\to 2}=\frac{{\omega \mu }_{0}}{32\pi d}D_{1}\cdot D_{2}\;\;\;\;\;\;\;\;\;\;\;\;\;Units:\;\frac{1}{sec\;m}\frac{V\;{sec}^{2}}{q\;m}\frac{q\;m}{sec}\frac{q\;m}{sec}=\frac{q\;V}{sec}$$

So, the energy $E_{hc}$ transferred in one half-cycle π/ω will be:

(28) $${\;\;\;\;\;\;\;\;\;\;\;\;E_{hc}=\frac{\pi }{\omega }P}_{1\to 2}=\frac{\mu_{0}}{32\;d}D_{1}\cdot D_{2}\;\;\;\;\;\;\;\;\;\;\;\;\;Units:\;\frac{1}{m}\frac{V\;{sec}^{2}}{q\;m}\frac{q\;m}{sec}\frac{q\;m}{sec}=q\;V$$

Now, let us imagine that the energy is being transmitted by an “$\vec{a}$-field” and absorbed by a “$\vec{j}$-field” but both of these fields are *potentials*—they have no energy of their own, but the first is the retarded potential of the Emitter dipole $D_{1}$ and the second is the advanced potential of the Absorber dipole $D_{2}$. These potentials radiate outward at velocity ±c and their amplitude decreases with distance in such a way that the total energy in their product, integrated over a half-wavelength, gives the total energy in Equation (28).

In order to do this integral, we need to estimate the area of the region of overlap in which the two signals are in phase. As in [3], we judge that paths longer than d+λ/4 will average to zero. In Section 3, we will find that this assumption needs to be revised at large distances. We calculate the “width” w of the region of interest that has less than that extra delay. The “length” l of the path, from the Emitter to a point x=r, y=w and thence to the Absorber, with the approximation that w≪d:

(29) $$l\left(r\right)\approx \sqrt{r^{2}+w^{2}}+\sqrt{{\left(d-r\right)}^{2}+w^{2}}\approx d+\frac{{d\;w}^{2}}{2\left(d-r\right)r}=\;d+\frac{\lambda }{4}$$

(30) $$\frac{{d\;w}^{2}}{2\left(d-r\right)r}=\frac{\lambda }{4}\;\;\;\;\;\;\to \;\;\;\;\;\;w^{2}\approx \frac{1}{2}\left(1-\frac{r}{d}\right)\left(\frac{r}{d}\right)d\;\lambda$$

The volume ${Vol}_{half}$ of one half-wavelength “in flight” is then approximately:

(31) $${Vol}_{half}\approx {\pi w}^{2}\frac{\lambda }{2}\approx \frac{\pi }{4}\left(1-\frac{r}{d}\right)\left(\frac{r}{d}\right)d\;\lambda^{2}$$

The energy $E_{hc}$ in a half-cycle from Equation (28) divided by ${Vol}_{half}$ will be the energy density $\rho_{E}$ in the “overlap” of the vector potentials of the two atoms.

(32) $$E_{hc}=\frac{\mu_{0}}{32\;d}{\vec{D}}_{1}\cdot {\vec{D}}_{2}\;\approx \rho_{E}\frac{\pi }{4}\left(1-\frac{r}{d}\right)\left(\frac{r}{d}\right)d\;\lambda^{2}\;\;\;\;\;\;\;\;\;\;\;Units:\;\frac{1}{m}\frac{V\;{sec}^{2}}{q\;m}\frac{q\;m}{sec}\frac{q\;m}{sec}=q\;V\;\rho_{E}\approx \;\frac{\mu_{0}}{8\pi \lambda^{2}}\frac{{\vec{D}}_{1}}{r}\cdot \frac{{\vec{D}}_{2}}{d-r}\;\;\;\;\;\;\;\;Units:\;\frac{1}{m^{2}}\frac{V\;{sec}^{2}}{q\;m}\frac{q\;m}{m\;sec}\frac{q\;m}{m\;sec}=\frac{q\;V}{m^{3}}$$

In this special case, where d≫λ, we have shown that thinking of the energy as located in the overlap of the Emitter’s retarded vector potential and the Absorber’s advanced vector potential gives the same result as the known Green’s Function action-at-a-distance view put forth in [3].

**Figure 5:**
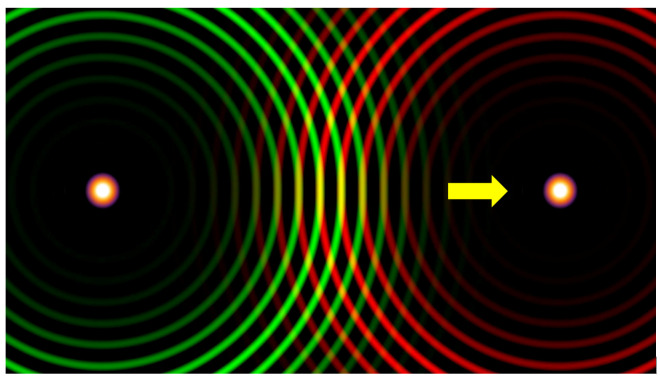
The interaction between the Emitter and Absorber vector potential waves. The waves are in phase. This is the condition for a successful photon. This wave can be seen as an animation in [19].

The Emitter and Absorber vector potential waves are shown together in Figure 5.

## 3. Photon Energy in the RMS Product of the Emitter and Absorber Waves

We now consider the Product P(x,t), which is proportional to the product of the Emitter and Absorber vector potentials, but has the dimensions of an energy density in units of $\frac{q\;V}{m^{3}}$.

(33) $$P\left(x,t\right)\propto \;A_{{⊥}_{E}}\left(x,t\right)\cdot A_{{⊥}_{A}}\left(x,t\right)$$

(34) $$P\left(x,t\right)=C{\left(\frac{\mu_{0}\omega^{2}D_{0}}{4\pi }\right)}^{2}\frac{sin\left(\theta \right)cos\left(\omega t_{E}\right)sin\left(\theta_{A}\right)cos\left(\omega t_{A}+\varphi \right)\;\hat{\theta }\cdot {\hat{\theta }}_{A}}{r\;\left(e^{t_{E}/\tau }+1\right)\left(e^{-t_{E}/\tau }+1\right)r_{A}\;\left(e^{t_{A}/\tau }+1\right)\left(e^{-t_{A}/\tau }+1\right)}$$

(35) $$P\left(x,t\right)=C\frac{{\mu_{0}}^{2}\omega^{4}{D_{0}}^{2}}{16\pi^{2}}\frac{sin\left(\theta \right)cos\left(\omega t_{E}\right)sin\left(\theta_{A}\right)cos\left(\omega t_{A}+\varphi \right)\;\hat{\theta }\cdot {\hat{\theta }}_{A}}{r\;\left(e^{t_{E}/\tau }+1\right)\left(e^{-t_{E}/\tau }+1\right)r_{A}\;\left(e^{t_{A}/\tau }+1\right)\left(e^{-t_{A}/\tau }+1\right)}$$

$$Units:\;\;\frac{q\;{sec}^{2}}{m\;V}{\left(\frac{V\;{sec}^{2}}{q\;m}\right)}^{2}\frac{1}{{sec}^{4}}{\left(\frac{q\;m}{sec}\right)}^{2}\frac{1}{m^{2}}=\frac{q\;V}{m^{3}}$$

where C has units of $\frac{q\;{sec}^{2}}{m\;V}$, i.e., $\frac{Coulomb\;{second}^{2}}{Volt\;meter}$ or $\frac{Farad\;{second}^{2}}{meter}$, and where $t_{E}$, $r_{A}$ and $t_{A}$ are defined in Equations (21), (24), and (25), respectively, and we have used

(36) $$\hat{z}\cdot \hat{\theta }=sin\left(\theta \right)\;\;\;\;\;\;\;\;\;\;\;\;\;\;\;\;\;\hat{z}\cdot {\hat{\theta }}_{A}=sin\left(\theta_{A}\right)\;\;\;\;\;\;\;\;\;\;\;\;\;\;\;\lambda =\frac{2\pi c}{\omega }$$

C is necessary to establish the correct conversion scaling from the product of vector potentials to energy density.

**Figure 6:**
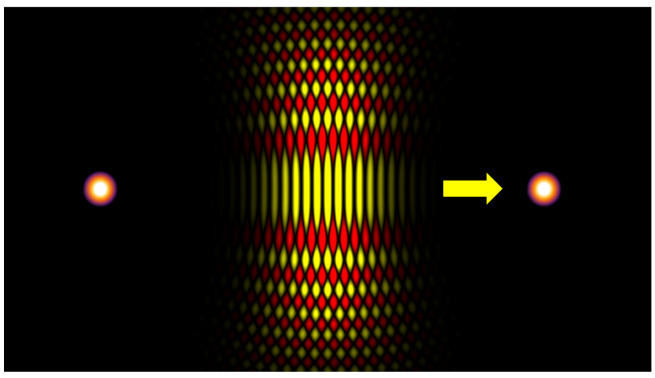
The Signed Product P(x,t) of the Emitter and Absorber vector potential waves, showing the photon energy wavepacket in flight at the speed of light from Emitter to Absorber at the halfway point through the transfer, as seen in the *x*–*y* plane. Yellow indicates positive sign of the product, red indicates negative sign of the product. The waves are in phase, so the main large central product wave-train is positive (yellow), and the total integrated product is positive, leading to a net positive energy transfer from Emitter to Absorber. This is the condition for a successful photon. This wave can be seen as an animation in [19].

The Product P(x,t) of the Emitter and Absorber vector potential waves are shown together in Figure 6.

Now we will describe the RMS operation as a natural part of computing the power in an oscillatory wave, i.e., we are not proposing some new mystical rectifier operation that we have to justify physically, we are just computing the RMS power in the oscillatory product wave P(x,t). Here is the process to compute the Total Energy in Transit.

We will define the Total Energy in Transit E(t) at a given time t as the RMS integral of the energy density P(x,t) over all space, and over a half temporal cycle:

(37) $$E\left(t\right)=\int \sqrt{\;\;\frac{2}{T}\int_{t}^{t+T/2}{P\left(x,t\right)}^{2}\;dt}\;dvol$$

(38) $$E\left(t\right)=C\frac{{\mu_{0}}^{2}\omega^{4}{D_{0}}^{2}}{16\pi^{2}}\int \sqrt{\frac{2}{T}\int_{t}^{t+T/2}{\left(\frac{sin\left(\theta \right)cos\left(\omega t_{E}\right)sin\left(\theta_{A}\right)cos\left(\omega t_{A}+\varphi \right)\;\hat{\theta }\cdot {\hat{\theta }}_{A}}{r\;\left(e^{t_{E}/\tau }+1\right)\left(e^{-t_{E}/\tau }+1\right)r_{A}\;\left(e^{t_{A}/\tau }+1\right)\left(e^{-t_{A}/\tau }+1\right)}\right)}^{2}\;dt}\;dvol$$

(39) $$Units:\;\frac{q\;{sec}^{2}}{m\;V}{\left(\frac{V\;{sec}^{2}}{q\;m}\right)}^{2}\frac{1}{{sec}^{4}}{\left(\frac{q\;m}{sec}\right)}^{2}\frac{m^{3}}{m^{2}}=q\;V$$

The Total Energy in Transit reduces to the following expression, as shown in Appendix A:

(40) $$E\left(t\right)=C\frac{{\mu_{0}}^{2}\omega^{4}{D_{0}}^{2}}{32\pi^{2}}\int \frac{\sqrt{\;1+\frac{1}{2}cos\left(2k\left(r+r_{A}\right)+2\varphi \right)}}{r\;\left(e^{t_{E}/\tau }+1\right)\left(e^{-t_{E}/\tau }+1\right)r_{A}\;\left(e^{t_{A}/\tau }+1\right)\left(e^{-t_{A}/\tau }+1\right)}\;dvol$$

In terms of the spatial variables only, the degree of progression of the photon from Emitter to Absorber is

(41) $$\xi =\frac{x}{d}$$

And the function to be integrated at a given value of ξ is

(42) $$E\left(\xi \right)=C\frac{{\mu_{0}}^{2}\omega^{4}{D_{0}}^{2}}{32\pi^{2}}\int \frac{\sqrt{\;1+\frac{1}{2}cos\left(\frac{2k}{c\tau }\left(r_{E}+r_{A}\right)\right)}}{r_{E}\;r_{A}\;\left(e^{\frac{r_{EE}}{c\tau }}+1\right)\left(e^{-\frac{r_{EE}}{c\tau }}+1\right)\left(e^{\frac{r_{AA}}{c\tau }}+1\right)\left(e^{-\frac{r_{AA}}{c\tau }}+1\right)}\;dvol$$

where we are now using k= ωτ as a dimensionless constant, which determines how many dipole wave cycles are in a photon wavepacket, and

(43) $$r_{E}=\sqrt{x^{2}+y^{2}+z^{2}}\;\;\;\;\;\;\;\;\;\;\;\;\;\;\;\;r_{A}=\sqrt{{\left(d-x\right)}^{2}+y^{2}+z^{2}}$$

are the distances from any point in space to the Emitter and Absorber Atoms, respectively, and

(44) $$r_{EE}=r_{E}-\xi d\;\;\;\;\;\;\;\;\;\;\;\;\;\;\;\;r_{AA}=r_{A}-\left(1-\xi \right)d$$

are the distances from any point in space to the sphere representing the center of the Emitter and Absorber pulses, respectively.

**Figure 7:**
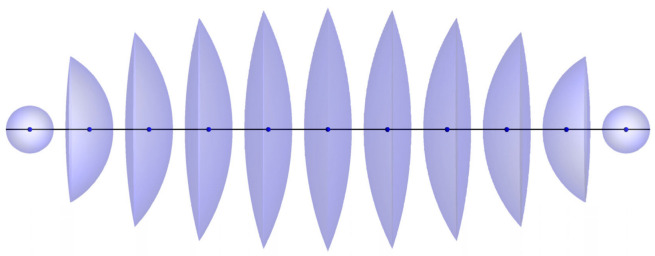
The lens-shaped volume as a function of degree of progression ξ, from 0 to 1 in steps of 0.1. The argument of the integral in Equation (42) is negligible outside of the volume, for each value of ξ. The width of the lens-shaped volume is determined by the parameter τ.

The photon energy is localized in the lens-shaped intersection region of the product P(x,t) of the Emitter and Absorber vector potential waves, which move from the Emitter to the Absorber Atom as a function of ξ from 0 to 1, as shown in Figure 7.

**Figure 8:**
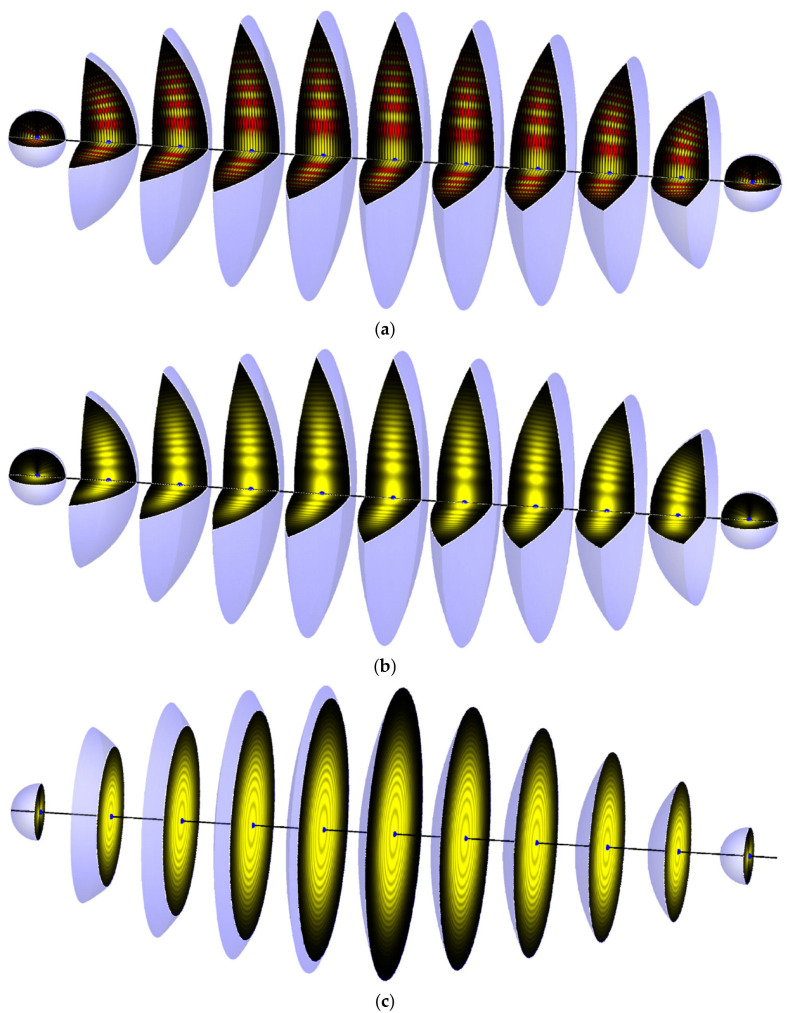
(**a**) P(x,t) from Equation (35) in the Cosine Phase condition, as a function of ξ, from 0 to 1 in steps of 0.1. Yellow represents positive values, red represents negative values. (**b**) RMS Energy Density from Equation (42). (**c**) RMS Energy Density from Equation (42).

The integrands in Equations (35) and (42) are shown in Figure 8.

**Figure 9:**
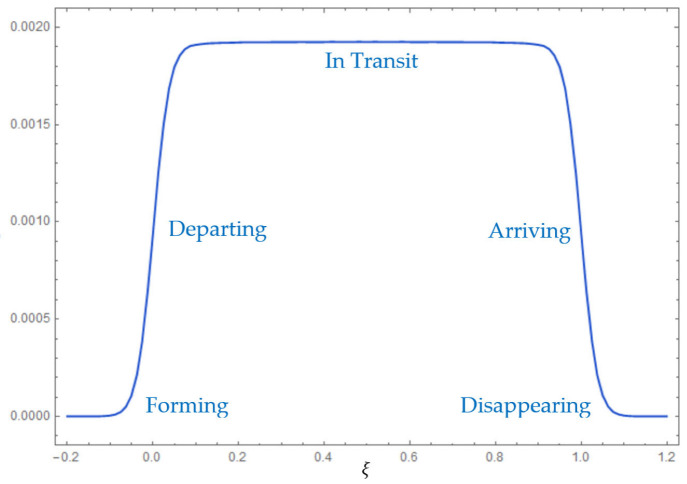
E(ξ) from Equation (42) in the Cosine Phase condition, as a function of ξ, from 0 to 1 in steps of 0.1, for d=1,cτ=0.025,k=8. Notice that the Energy in Transit is constant, independent of r.

We show the expected behavior using numerical integration in Figure 9.

### 3.1. Closed-Form Solution for the Photon Energy

We now proceed with the analytic integration of Equation (42):

(45) $$E\left(\xi \right)=C\frac{{\mu_{0}}^{2}\omega^{4}{D_{0}}^{2}}{32\pi^{2}}\int \frac{\sqrt{\;1+\frac{1}{2}cos\left(\frac{2k}{c\tau }\left(r_{E}+r_{A}\right)\right)}}{r_{E}\;r_{A}\;\left(e^{\frac{r_{EE}}{c\tau }}+1\right)\left(e^{-\frac{r_{EE}}{c\tau }}+1\right)\left(e^{\frac{r_{AA}}{c\tau }}+1\right)\left(e^{-\frac{r_{AA}}{c\tau }}+1\right)}\;dvol$$

The details of the lengthy calculation are given in Appendix B. We can determine that the Total Energy in Transit at the halfway point between the Emitter and Absorber is

(46) $$E(\frac{1}{2})\approx C\frac{{\mu_{0}}^{2}\omega^{4}{D_{0}}^{2}}{32\pi^{2}}\frac{1}{4d^{2}}\int \left(\frac{63}{64}+\frac{1}{4}cos\left(\frac{2k}{c\tau }\left(\frac{3x^{2}+{4y}^{2}+4z^{2}}{2d}\right)\right)-\frac{1}{64}\left(cos\left(\frac{4k}{c\tau }\left(\frac{3x^{2}+{4y}^{2}+4z^{2}}{2d}\right)\right)\right)\right)e^{-\frac{9\pi \left(\frac{y^{4}+z^{4}}{d^{2}}+x^{2}\right)}{64{\left(c\tau \right)}^{2}}}\;dvol$$

The high-frequency oscillatory terms make a zero net contribution to the integral, so we have

(47) $$E(\frac{1}{2})\approx C\frac{{\mu_{0}}^{2}\omega^{4}{D_{0}}^{2}}{32\pi^{2}}\frac{1}{4d^{2}}\int \left(\frac{63}{64}\right)e^{-\frac{9\pi \left(\frac{y^{4}+z^{4}}{d^{2}}+x^{2}\right)}{64{\left(c\tau \right)}^{2}}}\;dvol$$

(48) $$E(\frac{1}{2})\approx C\frac{{\mu_{0}}^{2}\omega^{4}{D_{0}}^{2}}{32\pi^{2}}\left(\frac{63}{64}\right)\frac{1}{4d^{2}}\int e^{-\frac{9\pi \left(\frac{y^{4}+z^{4}}{d^{2}}+x^{2}\right)}{64{\left(c\tau \right)}^{2}}}\;dvol$$

(49) $$E(\frac{1}{2})\approx C\frac{{\mu_{0}}^{2}\omega^{4}{D_{0}}^{2}}{32\pi^{2}}\left(\frac{63}{64}\right)\left(-\frac{\Gamma \left(-\frac{3}{4}\right)\Gamma \left(\frac{1}{4}\right)}{3\sqrt{\pi }\;}\frac{{\left(c\tau \right)}^{2}}{d\;}\right)$$

Therefore,

(50) $$E(\frac{1}{2})\approx C\frac{{\mu_{0}}^{2}\omega^{4}{D_{0}}^{2}}{32\pi^{2}}\left(-\frac{63\Gamma \left(-\frac{3}{4}\right)\Gamma \left(\frac{1}{4}\right)}{192\sqrt{\pi }\;}\right)\frac{{\left(c\tau \right)}^{2}}{d\;}$$

Evaluating this unfamiliar factor:

(51) $$\left(-\frac{63\Gamma \left(-\frac{3}{4}\right)\Gamma \left(\frac{1}{4}\right)}{192\sqrt{\pi }\;}\right)=3.24463=1.0328\;\pi$$

At d=1,cτ=0.025,k=8, we find that $E\left(\frac{1}{2}\right)=C\frac{{\mu_{0}}^{2}\omega^{4}{D_{0}}^{2}}{32\pi^{2}}\left(0.00202789\right)$. The numerical result shown in Figure 9 gives $\left(\frac{1}{2}\right)=C\frac{{\mu_{0}}^{2}\omega^{4}{D_{0}}^{2}}{32\pi^{2}}\left(0.00192484\right)$. So the analytic result is too large by a factor of $\frac{0.00202789}{0.00192484}=1.05354$—about 5.4% too big. Of course, the analytic result contains many approximations, each of which can contribute an error of 1–2%, and these approximation errors may accumulate.

A corrected result would have a value for the constant factor of $\frac{3.24463}{1.05354}=3.07974\approx \frac{3+\pi }{2}=3.0708$. Therefore, we propose the following as an improved approximation:

(52) $$E(\frac{1}{2})\approx C\frac{{\mu_{0}}^{2}\omega^{4}{D_{0}}^{2}}{32\pi^{2}}\left(\frac{3+\pi }{2}\right)\frac{{\left(c\tau \right)}^{2}}{d\;}$$

At d=1,cτ=0.025,k=8, we find that $E\left(\frac{1}{2}\right)=C\frac{{\mu_{0}}^{2}\omega^{4}{D_{0}}^{2}}{32\pi^{2}}\left(0.00191925\right)$, in agreement within 0.3% with the numerical result shown in Figure 9.

**Figure 10:**
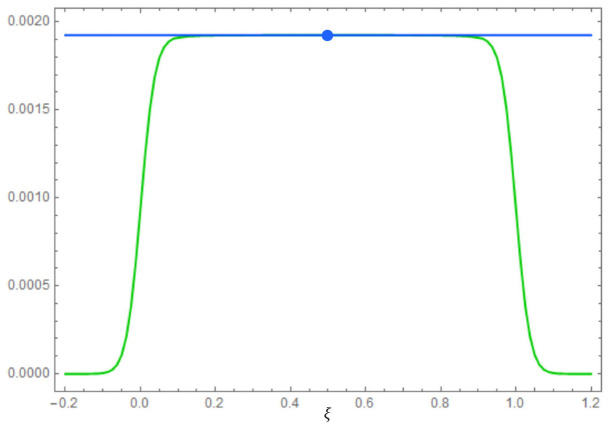
Sine Phase steady-state value $E\left(\frac{1}{2}\right)$ (blue) and the full numerical result (green) for d=1,cτ=0.025,k=8.

Now we have an approximate analytic model for the RMS steady-state energy value, shown in Figure 10 with d=1,cτ=0.025,k=8.

**Figure 11:**
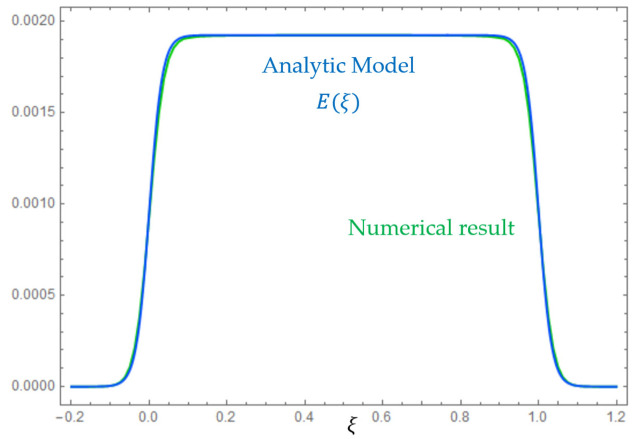
Sine Phase Analytical Model E(ξ) and the full numerical result for d=1,cτ=0.025,k=8.

Now we will model the entire rising and falling phases of the photon with symmetrical hyperbolic tangent functions:

(53) $$E\left(\xi \right)=C\frac{{\mu_{0}}^{2}\omega^{4}{D_{0}}^{2}}{32\pi^{2}}\left(\frac{3+\pi }{2}\right)\frac{{\left(c\tau \right)}^{2}}{d\;}\left(\frac{1+tanh\left(\beta \xi \right)}{2}\right)\left(\frac{1+tanh\left(\beta \left(1-\xi \right)\right)}{2}\right)$$

as shown in Figure 11.

We find an excellent fit with β=30 for d=1,cτ=0.025,k=8. We expect β to depend inversely on cτ and directly on d, so we propose:

(54) $$\beta =\;\frac{\;3d}{4c\tau \;}$$

Therefore,

(55) $$E\left(\xi \right)=C\frac{{\mu_{0}}^{2}\omega^{4}{D_{0}}^{2}}{32\pi^{2}}\left(\frac{3+\pi }{2}\right)\frac{{\left(c\tau \right)}^{2}}{d\;}\left(\frac{1+tanh\left(\frac{3d\xi }{4c\tau }\right)}{2}\right)\left(\frac{1+tanh\left(\frac{3d\left(1-\xi \right)}{4c\tau }\right)}{2}\right)$$

(56) $$Units:\frac{q\;{sec}^{2}}{m\;V}{\left(\frac{V\;{sec}^{2}}{q\;m}\right)}^{2}\frac{1}{{sec}^{4}}{\left(\frac{q\;m}{sec}\right)}^{2}\frac{m^{2}}{m}=q\;V$$

### 3.2. Lossless Energy Transmission over Long Distances

**Figure 12:**
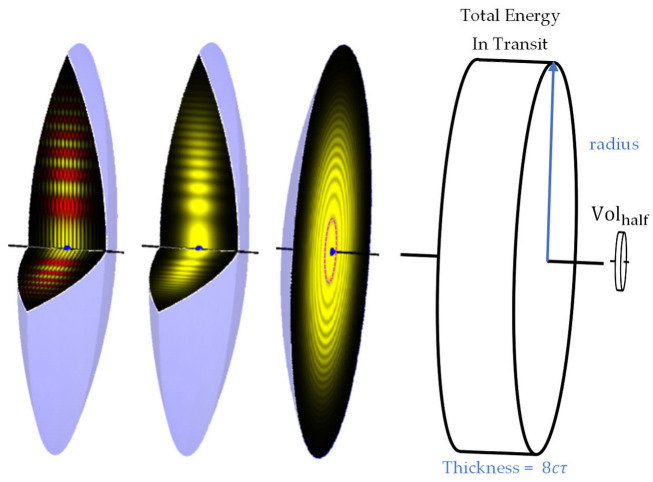
Cylindrical approximation to the lens-shaped volume for the Total Energy in Transit (large cylinder). The smaller cylinder represents the ${Vol}_{half}$ region described in Section 4. The radius of ${Vol}_{half}$ corresponds to the dotted magenta line in the *y*–*z* slice, at the first amplitude null in the Cosine Phase configuration at ¼ of a wave cycle.

Now we consider the scaling of E(t) with r. The non-oscillatory r-dependent terms in Equation (42) represent the volume of the photon scaled by the 1/r distances to the two atoms:

(57) $$E\left(t\right)\propto \int \frac{1}{r_{E}\;\left(e^{t_{E}/\tau }+1\right)\left(e^{-t_{E}/\tau }+1\right)r_{A}\;\left(e^{t_{A}/\tau }+1\right)\left(e^{-t_{A}/\tau }+1\right)}\;dvol$$

(58) $$\approx \frac{1}{r_{E}\;r_{A}}\int \frac{1}{\left(e^{t_{E}/\tau }+1\right)\left(e^{-t_{E}/\tau }+1\right)\left(e^{t_{A}/\tau }+1\right)\left(e^{-t_{A}/\tau }+1\right)}\;dvol$$

where the $\frac{1}{\left(e^{t_{E}/\tau }+1\right)\left(e^{-t_{E}/\tau }+1\right)\left(e^{t_{A}/\tau }+1\right)\left(e^{-t_{A}/\tau }+1\right)}$ term defines the lens-shaped volume at a given r—it is the multiplicative overlap of the two spherical pulses. To a good first approximation, we can treat the lens-shaped volume as proportional to the volume of an enclosing cylinder, as shown in Figure 12.

**Figure 13:**
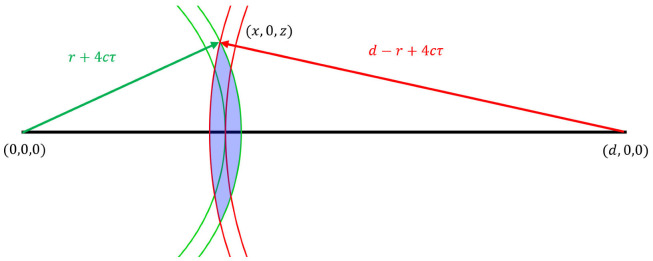
Finding the radius *z* of the lens (blue) in the *x*–*z* plane, for the photon in transit.

The thickness of the cylinder is the spatial length of the Dipole Envelope pulse $D_{e}\left(t\right)$ from Equations (2) and (21), which we can reasonably take to be 8cτ. So the thickness does not depend on r. But the radius of the lens does depend on r, as can be seen in Figure 8. The radius of the lens comes from the intersection of two spheres, one centered at the Emitter (0,0,0) with a radius of r+4cτ, the other centered at the Absorber (d,0,0) with a radius of d−r+4cτ. We can work the problem in two dimensions in the *x*–*z* plane, as shown in Figure 13.

(59) $$x^{2}+z^{\;2}={\left(r+4c\tau \right)}^{2}$$

(60) $${\left(d-x\right)}^{2}+z^{\;2}={\left(d-r+4c\tau \right)}^{2}$$

(61) $$d^{2}-2xd+x^{2}+z^{\;2}={\left(d-r+4c\tau \right)}^{2}$$

(62) $$d^{2}-2xd={\left(d-r+4c\tau \right)}^{2}-{\left(r+4c\tau \right)}^{2}$$

(63) $$x=\frac{1}{2d}\left(d^{2}-{\left(d-r+4c\tau \right)}^{2}+{\left(r+4c\tau \right)}^{2}\right)$$

(64) $$x=r-4c\tau \;\left(1-\frac{2r}{d}\right)$$

(65) $$z^{\;2}={\left(r+4c\tau \right)}^{2}-x^{\;2}$$

(66) $$z^{\;2}={\left(r+4c\tau \right)}^{2}-{\left(r-4c\tau \;\left(1-\frac{2r}{d}\right)\right)}^{2}$$

which miraculously simplifies to

(67) $$z^{\;2}=\frac{4\;\;}{d^{2}\;}\left(4c\tau \right)\left(d+4c\tau \right)\left(d-r\right)r$$

Now we can compute the volume of the lens-sized cylinder:

(68) $$E\left(t\right)\propto \;\frac{\pi \;thickness\;{radius}^{2}}{r_{E}\;r_{A}}$$

(69) $$E\left(t\right)\propto \;\frac{\pi \;8c\tau \;z^{2}}{r_{E}\;r_{A}}$$

and using $z^{\;2}$ from Equation (67):

(70) $$E\left(t\right)\propto \;\frac{8\pi c\tau }{r\left(d-r\right)}\;\frac{4\;\;}{d^{2}\;}\left(4c\tau \right)\left(d+4c\tau \right)\left(d-r\right)r$$

All of the radial terms r(d−r) miraculously cancel out, leaving

(71) $$E\left(t\right)\propto \;\frac{8\pi \;\;}{d^{2}\;}{\left(4c\tau \right)}^{2}\left(d+4c\tau \right)$$

which is independent of *r*! Thus, the Total Energy in Transit is conserved as the photon travels from the Emitter to the Absorber. This remarkable, essential result arises directly from the 1/r dependence of the vector potentials and the geometry of the multiplicatively intersecting spherical pulses. This applies in the steady-state region 4cτ<r<d−4cτ, i.e., after the photon has left the Emitter, and before it has arrived at the Absorber.

### 3.3. Efficiency and the Path Fraction

From Appendix B, the $\sqrt{\;1+\frac{1}{2}cos\left(\frac{2k}{c\tau }\left(\frac{3x^{2}+{4y}^{2}+4z^{2}}{2d}\right)\right)}$ term in Equation (A57) gave rise to the Path Fraction S(k) in Equation (A58):

(72) $$S\left(k\right)\approx \frac{63}{64}$$

This means that about 98.4% of the energy in the lens-shaped product region is participating in the Total Energy in Transit of the photon! Here, we will give a physical interpretation of this phenomenon.

**Figure 14:**
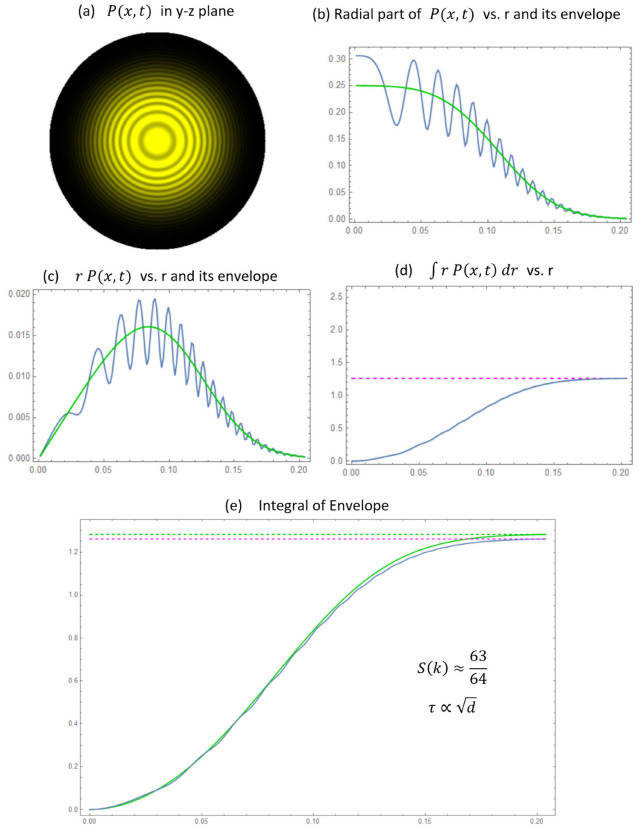
Visualizing the Path Fraction S(k) for the RMS Photon Model. (**a**) Cross-section of the photon P(x,t) at the halfway point in transit $\xi =\frac{1}{2}$. (**b**) The radial part of P(x,t). (**c**) rP(x,t). (**d**) The integral ∫r P(x,t) dr. (**e**) The integral of the envelope. S(k) is the ratio of ∫r P(x,t) dr over the integral of the envelope, i.e., the usable fraction of the available energy. In this example, $\;S\left(k\right)=\frac{1.2613}{1.2827}=0.9833$.

Consider the cross-section slice of the photon P(x,t) RMS Energy Density from Figure 8c, at the halfway point in transit $\xi =\frac{1}{2}$, as shown in Figure 14a. Notice the yellow (positive) rings of energy density. We can calculate the net positive contribution as illustrated in Figure 14b through Figure 14e. In Figure 14b, we see the radial part of the RMS Energy Density and its envelope. To compute the total integrated area in the circular region, we first multiply by r in Figure 14c, and then integrate in Figure 14d to get ∫r P(x,t) dr. The final integrated value in Figure 14d is marked by the horizontal dotted magenta line. In Figure 14e, we show the integral of the envelope (green), which reaches a slightly higher value. Now we can see graphically that the high-frequency oscillatory terms contribute very little to the integral. S(k) is the ratio of ∫r P(x,t) dr to the integral of the envelope, which turns out to be about 98.4% when full 3D integrals are performed.

### 3.4. Realistic Parameters

We now consider a realistic set of parameters for Hydrogen Atoms separated by a distance d=1 m, exchanging a photon of energy with the Lyman-Alpha <210> to <100> transition.

The following physical constants will be useful:

- Speed of light:           c=299,792,458 m/s.
- Lyman-Alpha frequency:       $f_{21}=2.46604\times 10^{15}\;Hz.$
- Lyman-Alpha angular frequency: $\omega_{21}=1.54946\times 10^{16}\;rad/s$.
- Lyman-Alpha wavelength:       $\lambda_{21}=1.21568\times 10^{-7}\;m$.
- Lyman-Alpha energy:         $E_{21}=1.63401\times 10^{-18}\;J=10.1987\;eV$.
- Bohr radius:              $a_{0}=5.29465\times 10^{-11}\;m$.
- Electron charge:          $q_{e}=1.60217653\times 10^{-19}\;C.$
- Magnetic constant:        $\mu_{0}=4\pi \times 10^{-7}\;N{/A}^{2}$.

We need to determine τ. From Equation (55), the Total Energy in Transit is determined, and by conservation of energy, it can be set equal to the Emitter transition energy $E_{21}$:

(73) $$E\left(\xi =\frac{1}{2}\right)=\;C\frac{{\mu_{0}}^{2}\omega^{4}{D_{0}}^{2}}{32\pi^{2}}\left(\frac{3+\pi }{2}\right)\frac{{\left(c\tau \right)}^{2}}{d\;}=E_{21}$$

(74) $$Units:\;\;\frac{q\;{sec}^{2}}{m\;V}{\left(\frac{V\;{sec}^{2}}{q\;m}\right)}^{2}\frac{1}{{sec}^{4}}{\left(\frac{q\;m}{sec}\right)}^{2}\frac{m^{2}}{m}=q\;V$$

Therefore,

(75) $$\tau =\sqrt{E_{21}\frac{64\pi^{2}d}{C{{\left(3+\pi \right)\mu }_{0}}^{2}\omega^{4}{D_{0}}^{2}c^{2}}}\;\;\;\;\;\;\;\;\;\;\;⟹\;\;\;\;\;\;\;\;\;\;\tau \propto \sqrt{d}$$

which depends on C. In the next section, we will develop a principled method for computing C, which will give the following final value:

(76) $$C=3.82057\times 10^{-30}\;\frac{F\;{sec}^{2}}{m}$$

Now, we will use this final value of C to consider five other values of d:

- The distance to the Sun (150 billion meters).
- One light year.
- The faintest distant visible star, HD 92740 [24]. This is the most distant Blue star with an apparent magnitude of 6.5 in the SIMBAD database (just barely visible to the human eye), with a distance of 2565.418 parsecs.
- The most distant visible celestial object, the Andromeda Galaxy [25].
- The most distant known object, JADES-GS-z13-0 [26].

**Figure 15:**
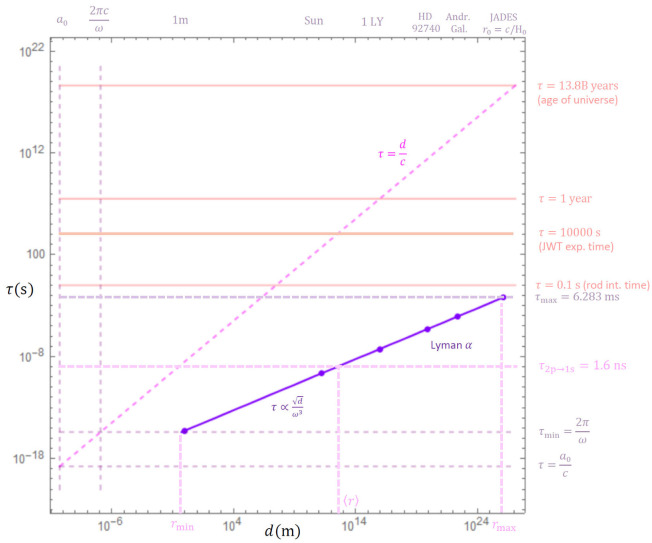
The relationship of transition time constant τ to the Emitter–Absorber distance d, with some familiar reference points.

We summarize the results below in Table 1:

In Figure 15, we show the relationship between the transition time constant τ and the Emitter–Absorber distance d, with some familiar reference points.

## 4. Proportionality Between RMS Product of Vector Potential Waves and Energy Density

Our approach to determining the proportionality constant C will be based on these key insights:

1. From Fermi’s Golden Rule, the mean transition time $\tau_{2p\to 1s}$ for the Hydrogen Lyman-Alpha transition is about 1.6 ns, as shown in Figure 15.
2. Fermi’s Golden Rule integrates the contributions over all of the modes of the vacuum to find the mean transition time $\tau_{2p\to 1s}$
3. In our framework, using Mach’s Principle, we will derive the mean transition time 〈τ(d)〉 by integrating the weighted contributions of the model over all of the potential Absorbers in the universe, i.e., over the full length of the purple line in Figure 15, from $r_{min}$ to $r_{max}$.
4. The contributions of the potential Absorbers at distance d will be weighted by the number of Absorbers at that distance, times an attenuation factor.
5. By setting our mean transition time 〈τ(d)〉 equal to $\tau_{2p\to 1s}$ from Fermi’s Golden Rule, we can solve for C.

### 4.1. The Mean Lifetime of the Hydrogen Lyman-Alpha Transition from Fermi’s Golden Rule

First, we will establish the standard calculation of the mean lifetime of the Hydrogen Lyman-Alpha transition [27,28]. Beginning with the standard solutions of the Schrodinger Equation for the n=1 state and n=2 state, as shown in Table 2:

The Matrix Elements are

(77) $$\left\langle \psi_{1}|op|\psi_{2m}\right\rangle =\int \psi_{1}^{*}\;op\;\psi_{2m}r^{2}dr\;d\theta \;d\phi \;$$

We want

(78) $${\left|\left\langle \psi_{1}|r|\psi_{2m}\right\rangle \right|}^{2}={\left|\left\langle \psi_{1}|x|\psi_{2m}\right\rangle \right|}^{2}+{\left|\left\langle \psi_{1}|y|\psi_{2m}\right\rangle \right|}^{2}+{\left|\left\langle \psi_{1}|z|\psi_{2m}\right\rangle \right|}^{2}$$

(79) $$\left\langle \psi_{1}|x|\psi_{2m}\right\rangle =\frac{2^{15/2}}{3^{9/2}}\sqrt{\frac{1}{6}}a_{0}\left(\delta_{m,-1}-\delta_{m,1}\right)$$

(80) $$\left\langle \psi_{1}|y|\psi_{2m}\right\rangle =\frac{2^{15/2}}{3^{9/2}}\sqrt{\frac{1}{6}}a_{0}\left(\delta_{m,-1}+\delta_{m,1}\right)$$

(81) $$\left\langle \psi_{1}|z|\psi_{2m}\right\rangle =\frac{2^{15/2}}{3^{9/2}}\sqrt{\frac{1}{3}}a_{0}\delta_{m,0}$$

Therefore,

(82) $$r_{12}^{2}={\left|\left\langle \psi_{1}|r|\psi_{2m}\right\rangle \right|}^{2\;}={\left(\frac{2^{15/2}}{3^{9/2}}a_{0}\right)}^{2}\frac{1}{3}=\frac{2^{15}}{3^{10}}a_{0}^{2}=\frac{32,768}{59,049}{\;a}_{0}^{2}\;\approx \;0.554929{\;a}_{0}^{2}$$

(83) $$r_{12}=\frac{2^{15/2}}{3^{10/2}}a_{0}=\sqrt{\frac{32,768}{59,049}}a_{0}\;\approx \;0.744936\;a_{0}$$

independent of the value of m. $r_{12}$ is a characteristic distance associated with the dipole created by the superposition of the two eigenstates. It seems reasonable that it is about ¾ of $a_{0}$.

Now we can determine the spontaneous transition rate (Einstein A value):

(84) $$w_{spon}\;\left(2p\to 1s\right)=\frac{4\omega^{3}}{3\hbar c^{3}}\;\frac{q^{2}}{4\pi \epsilon_{0}}r_{12}^{2}=\;6.3\times 10^{8}\;s^{-1}$$

And, therefore, the mean lifetime is

(85) $$\tau_{2p\to 1s}=\frac{1}{w_{spon}\;\left(2p\to 1s\right)}=1.5873\times 10^{-9}\;s$$

(86) $$\tau_{2p\to 1s}=\frac{\hbar c^{3}}{a_{0}^{2}\omega^{3}}\frac{\pi \epsilon_{0}}{q^{2}}\frac{3^{11}}{2^{15}}=\frac{177147}{32768}\frac{\hbar c^{3}}{a_{0}^{2}\omega^{3}}\frac{\pi \epsilon_{0}}{q^{2}}=1.5873\times 10^{-9}\;s$$

Now we can make some observations about $\tau_{2p\to 1s}$:

- $\tau_{2p\to 1s}=1.5873\times 10^{-9}\;s$ happens to be quite close to the RMS model value $\tau =2.5\times 10^{-9}s$ at the distance of the Sun.
- $\tau_{2p\to 1s}=1.5873\times 10^{-9}\;s$ happens to be at the midpoint on the logarithmic scale (geometric mean) of the range of values of τ for the RMS model, which ranges from about $10^{-18}s$ to about 0.1s. We will use that.

Recall that $\tau_{2p\to 1s}$ is interpreted as the mean lifetime of the Hydrogen Lyman-Alpha transition. In our time-symmetric Emitter/Absorber framework, we could take the mean τ value over all possible Absorbers in the universe on the light cone, weighted by the number of possible Absorbers at each distance.

Now, we will do the calculation carefully. We know two things:

From Equation (75):

(87) $$\tau \left(d\right)=\sqrt{E_{21}\frac{64\pi^{2}d}{C{{\left(3+\pi \right)\mu }_{0}}^{2}\omega^{4}{D_{0}}^{2}c^{2}}}⟹\tau \propto \sqrt{d}$$

And the mean value of τ(d) from Fermi’s Golden Rule in Equation (86) is

(88) $$\langle \tau \left(d\right)\rangle =\tau_{2p\to 1s}=\frac{\hbar c^{3}}{a_{0}^{2}\omega^{3}}\frac{\pi \epsilon_{0}}{q^{2}}\frac{3^{11}}{2^{15}}=\frac{177147}{32768}\frac{\hbar c^{3}}{a_{0}^{2}\omega^{3}}\frac{\pi \epsilon_{0}}{q^{2}}=1.5873\times 10^{-9}\;s$$

We want to find 〈τ(d)〉, assuming that the density of possible Absorbers D(r) is constant, and we expect to integrate over a range from ${r_{min}\approx 10}^{-6}m$ to ${r_{max}\approx 10}^{26}m$. And then with the expression for 〈τ(d)〉, we will be able to solve for the constant C.

After some experimentation, we found a good solution in the following form:

(89) $$\langle \tau \left(d\right)\rangle =\tau \left(\sqrt{r_{min}r_{max}}\right)$$

### 4.2. Determining the Proportionality Constant C

This will then give us a second equation involving τ and d, which we can then combine with Equation (87) for the RMS Photon Model to determine the missing constant of proportionality C. Combining Equations (87)–(89),

(90) $$\langle \tau \left(d\right)\rangle =\tau \left(\sqrt{r_{min}r_{max}}\right)=\sqrt{E_{21}\frac{64\pi^{2}\sqrt{r_{max}r_{min}}}{C{{\left(3+\pi \right)\mu }_{0}}^{2}\omega^{4}{D_{0}}^{2}c^{2}}}=\frac{177,147}{32,768}\frac{\hbar c^{3}}{a_{0}^{2}\omega^{3}}\frac{\pi \epsilon_{0}}{q^{2}}$$

(91) $$E_{21}\frac{64\pi^{2}\sqrt{r_{max}r_{min}}}{C{{\left(3+\pi \right)\mu }_{0}}^{2}\omega^{4}{D_{0}}^{2}c^{2}}={\left(\frac{177,147}{32,768}\frac{\hbar c^{3}}{a_{0}^{2}\omega^{3}}\frac{\pi \epsilon_{0}}{q^{2}}\right)}^{2}$$

And thus the constant of proportionality C is

(92) $$C=E_{21}\frac{64\pi^{2}\sqrt{r_{max}r_{min}}}{{{\left(3+\pi \right)\mu }_{0}}^{2}\omega^{4}{D_{0}}^{2}c^{2}}{\left(\frac{32,768}{177,147}\frac{a_{0}^{2}\omega^{3}}{\hbar c^{3}}\frac{q^{2}}{\pi \epsilon_{0}}\right)}^{2}$$

But this expression for C depends on $r_{max}$ and $r_{min}$.

To find $r_{max}$, let us consider the most distant object that has ever been observed in the universe, and compare it to the radius of the Hubble Sphere $r_{0}=c/H_{0}$, which is the distance to the most distant object that could ever be observed in an expanding universe.

The most distant object that has ever been observed in the universe, as of August 2023, is JADES-GS-z13-0, a high-redshift Lyman-break galaxy discovered by the James Webb Space Telescope (JWST) during NIRCam imaging for the JWST Advanced Deep Extragalactic Survey (JADES) on 29 September 2022 [29]. Relative to Earth, JADES-GS-z13-0 has a light-travel distance of 13.6 billion light-years, or $1.2867\times 10^{26}\;m$.To compute the radius of the Hubble Sphere $r_{0}=c/H_{0}$, we need the value of the Hubble Constant $H_{0}$, which is currently contested [30,31]. We will use the value

(93) $$H_{0}=72.94\;\pm 1.98\;km/s/Mpc=2.36358\times 10^{-18}\;s^{-1}$$

which comes from the Median of Variants of the Tip of the Red Giant Branch [31]. This leads to

(94) $$r_{0}=c/H_{0}=1.26838\times 10^{26}\;m$$

which is within 1.4% of the JADES-GS-z13-0 light travel distance of $1.2867\times 10^{26}\;m$. So, for $r_{max}$, we will use

(95) $$r_{max}=r_{0}=c/H_{0}=1.26838\times 10^{26}\;m$$

as shown in Figure 15. Substituting into Equation (93) for our constant of proportionality C, we have:

(96) $$C=E_{21}\frac{64\pi^{2}}{{{\left(3+\pi \right)\mu }_{0}}^{2}\omega^{4}{D_{0}}^{2}c^{2}}{\sqrt{\frac{c\;r_{min}}{H_{0}}}\left(\frac{32,768}{177,147}\frac{a_{0}^{2}\omega^{3}}{\hbar c^{3}}\frac{q^{2}}{\pi \epsilon_{0}}\right)}^{2}$$

To find $r_{min}$, we need the value of d at which our model line intersects with the τ=2π/ω line in Figure 15. We can use the expression for τ from Equation (75):

(97) $$\tau =\sqrt{E_{21}\frac{64\pi^{2}d}{C{{\left(3+\pi \right)\mu }_{0}}^{2}\omega^{4}{D_{0}}^{2}c^{2}}}$$

to get

(98) $$\tau_{min}=\frac{2\pi }{\omega }=\sqrt{E_{21}\frac{64\pi^{2}r_{min}}{C{{\left(3+\pi \right)\mu }_{0}}^{2}\omega^{4}{D_{0}}^{2}c^{2}}}$$

Squaring both sides:

(99) $$\frac{4\pi^{2}}{\omega^{2}}=E_{21}\frac{64\pi^{2}r_{min}}{C{{\left(3+\pi \right)\mu }_{0}}^{2}\omega^{4}{D_{0}}^{2}c^{2}}$$

Therefore,

(100) $$r_{min}=\frac{C{{\left(3+\pi \right)\mu }_{0}}^{2}\omega^{2}{D_{0}}^{2}c^{2}}{16\;E_{21}}$$

Note that Equations (96) and (100) are both equations with unknowns C and $r_{min}$. Now we can combine them to solve for C:

(101) $$C=E_{21}\frac{64\pi^{2}}{{{\left(3+\pi \right)\mu }_{0}}^{2}\omega^{4}{D_{0}}^{2}c^{2}}{\sqrt{\frac{c\;4\pi^{2}C{{\left(3+\pi \right)\mu }_{0}}^{2}\omega^{4}{D_{0}}^{2}c^{2}}{H_{0}\omega^{2}E_{21}64\pi^{2}}}\left(\frac{32,768}{177,147}\frac{a_{0}^{2}\omega^{3}}{\hbar c^{3}}\frac{q^{2}}{\pi \epsilon_{0}}\right)}^{2}$$

Factoring out the $\sqrt{C}$ from both sides gives

(102) $$\sqrt{C}=E_{21}\frac{64\pi^{2}}{{{\left(3+\pi \right)\mu }_{0}}^{2}\omega^{4}{D_{0}}^{2}c^{2}}{\sqrt{\frac{c\;4\pi^{2}{{\left(3+\pi \right)\mu }_{0}}^{2}\omega^{4}{D_{0}}^{2}c^{2}}{H_{0}\omega^{2}E_{21}64\pi^{2}}}\left(\frac{32,768}{177,147}\frac{a_{0}^{2}\omega^{3}}{\hbar c^{3}}\frac{q^{2}}{\pi \epsilon_{0}}\right)}^{2}$$

Squaring both sides gives

(103) $$C={\left(\frac{E_{21}64\pi^{2}}{{{\left(3+\pi \right)\mu }_{0}}^{2}\omega^{4}{D_{0}}^{2}c^{2}}\right)}^{2}{\frac{c\;4\pi^{2}{{\left(3+\pi \right)\mu }_{0}}^{2}\omega^{4}{D_{0}}^{2}c^{2}}{H_{0}\omega^{2}E_{21}64\pi^{2}}\left(\frac{32,768}{177,147}\frac{a_{0}^{2}\omega^{3}}{\hbar c^{3}}\frac{q^{2}}{\pi \epsilon_{0}}\right)}^{4}$$

And simplifying, we have

(104) $$C=\frac{E_{21}256\pi^{4}}{{{\left(3+\pi \right)\mu }_{0}}^{2}\omega^{6}{D_{0}}^{2}cH_{0}}{\left(\frac{32,768}{177,147}\frac{a_{0}^{2}\omega^{3}}{\hbar c^{3}}\frac{q^{2}}{\pi \epsilon_{0}}\right)}^{4}$$

which is an expression for C with no adjustable parameters. Note that it depends on the physical constants associated with electromagnetism (${q,\;\epsilon }_{0},\mu_{0}$), Quantum Mechanics ($q,\;\hbar ,a_{0}$), relativity (c) and gravity/cosmology ($H_{0}$).

Evaluating this expression for C gives

(105) $$C=3.82057\times 10^{-30}\;\frac{F\;{sec}^{2}}{m}$$

From Equation (98), we can compute $\tau_{min}$:

(106) $$\tau_{min}=\frac{2\pi }{\omega }=4.05508\times 10^{-16}\;s$$

And using the value of C from Equation (105) in Equation (87), with the expression for $r_{max}$ in Equation (95), we have the expression for $\tau_{max}$:

(107) $$\tau_{max}=\sqrt{E_{21}\frac{64\pi^{2}r_{max}}{C{{\left(3+\pi \right)\mu }_{0}}^{2}\omega^{4}{D_{0}}^{2}c^{2}}}=\sqrt{E_{21}\frac{64\pi^{2}}{C{{\left(3+\pi \right)\mu }_{0}}^{2}\omega^{4}{D_{0}}^{2}cH_{0}}}\;=\;6.283\;\mathrm{ms}$$

$\tau_{min}$ and $\tau_{max}$ are shown in Figure 15.

This is an important milestone in the development of our theory of the structure of the photon. We have matched Fermi’s Golden Rule, but instead of integrating over all the modes of the vacuum, we are integrating over all the possible Absorber interactions, which requires integrating over all Absorbers in the visible universe, out to the radius of the Hubble Sphere, accounting for the $1/\sqrt{r}$ weighting function for occlusion of distant objects by nearer objects. We have used Mach’s Principle, and it shows up in our expression for C with the appearance of the Hubble Constant $H_{0}$ in Equation (105).

And with the appearance of the Hubble Constant in our expression for the structure of the photon, we see that our theory for light from a Quantum Transition depends on cosmology, and therefore on gravity. This would appear to be the beginning of a direct unification of gravity, Quantum Theory, and electromagnetism (Collective Electrodynamics), brought together by Mach’s Principle.

## 5. Photon Arrival Rates

In Section 3.4 and Figure 15, we showed that the photon transition time constant τ was always less than $\tau_{max}=6.283\;\mathrm{ms}$, the value of τ for objects at the radius of the Hubble Sphere. Now we will check that the photon arrival rates are reasonable for two familiar astronomical objects, including the optical systems that are used to detect them:

- The faintest distant visible star, HD 92740 [24]. This is the most distant Blue star with an apparent magnitude of 6.5 in the SIMBAD database (just barely visible to the human eye), with a distance of 2565.418 parsecs. The optical system is the lens of the human eye with an area of approximately 1 square centimeter, and a rod integration time of about 0.1 s.
- The most distant known object, JADES-GS-z13-0 [26], with apparent magnitude of 29.43. The optical system is the James Webb Telescope, with a total reflector area of 25.4 square meters, and a maximum integration time of about 10,000 s, or about 2.8 h [29].

### 5.1. Photon Arrival Rate for Most Faint Distant Visible Star, HD 92740

We will calculate the photon arrival rate for the faintest distant visible blue star, HD 92740, with apparent magnitude 6.5, using a lens area of 1 square centimeter.

The photon arrival rate at the eye lens for HD 92740 is

(108) $${Photon\;Arrival\;Rate}_{HD92740}=\frac{{Flux}_{Blue}\;A\;c}{2.512^{M}\;\lambda \;E_{photonBlue}}=13738.3\frac{photons}{s}$$

where the standard flux ${Flux}_{Blue}=4.26\times 10^{-20}\;\mathrm{erg}/(s\;{cm}^{2}\;\mathrm{Hz})$ [23], $A=1\;{cm}^{2}$, apparent magnitude M=6.5, c=299,792,458 m/s, λ=470 nm, and $E_{photonBlue}=3.1\;eV=4.9668\times 10^{-19}\;J$.

Recall from Section 3.4 that the photon time constant for HD 92740 is $\tau =4.96\times 10^{-6}s$. And for the human eye, the rod integration time is 0.1 s, as shown in Figure 15. The total number of photons arriving in the rod integration time of 0.1 s is 1373.83 photons. Note that the photon time constant is short compared to the rod integration time, as required for successful detection of light.

### 5.2. Photon Arrival Rate for Most Distant Known Object, JADES-GS-z13-0

We will calculate the photon arrival rate for the most distant known object, JADES-GS-z13-0, with apparent magnitude 29.43, using the reflector area of the James Webb Telescope of 25.4 square meters.

The photon arrival rate at the James Webb Telescope reflector for JADES-GS-z13-0 is

(109) $${Photon\;Arrival\;Rate}_{JADES}=\frac{{Flux}_{Blue}\;A\;c}{2.512^{M}\;\lambda \;E_{photonBlue}}=2.3459\;\frac{photons}{s}\;$$

where the standard flux ${Flux}_{Blue}=4.26\times 10^{-20}\;\mathrm{erg}/(s\;{cm}^{2}\;\mathrm{Hz})$, $A=25.4\;m^{2}$, apparent magnitude M=29.43, c=299,792,458 m/s, λ=470 nm, and $E_{photonBlue}=3.1\;eV=4.9668\times 10^{-19}\;J$.

Recall from Section 3.4 that the photon time constant for JADES-GS-z13-0 is $\tau =6.328\times 10^{-3}s$. And for the James Webb Telescope, the maximum exposure time is 10,000 s or about 2.8 h. The total number of photons arriving in the maximum exposure time of 10,000 s is 23,459 photons. Note that the photon time constant is short compared to the exposure time, as required for successful detection of light.

## 6. Conclusions

In this paper, we have developed a time-symmetrical theory of energy exchange between two atoms, proposing a specific formulation of the interaction between Emitter and Absorber Atoms, in which the energy density is proportional to the root-mean-square of the product of retarded and advanced four-vector potential waves, and show how this interaction efficiently and completely transfers energy from the Emitter Atom to the Absorber Atom over arbitrary distances. We use Mach’s Principle to find the proportionality constant by matching the mean transition time constant for all possible Absorbers in the universe to the mean transition lifetime computed from Fermi’s Golden Rule, leading to a complete solution with no adjustable parameters. The solution represents the operation and internal wave structure of a photon, valid over 26 orders of magnitude in Emitter–Absorber distance d, from about 0.52 m to the radius of the Hubble Sphere $1.27\times 10^{26}m$.

The present theory differs from Cramer and Mead 2020 [3] in several important ways:

1. Cramer and Mead 2020 [3] proposed that energy density would be proportional to the sum of the Emitter and Absorber vector potentials, while the present paper proposed that energy density would be proportional to the root-mean-square of the product of the Emitter and Absorber vector potentials.
2. Cramer and Mead 2020 [3] did not explicitly account for the traveling pulse, producing a relationship τ∝d and, thus, it was valid only for small spacing d<0.52 m between the Emitter and Absorber Atoms. The current paper explicitly accounts for the traveling pulse, producing a relationship $\tau \propto \sqrt{d}$, which is valid for large spacing d>0.52 m between the Emitter and Absorber Atoms.
3. The combination of the above two differences allows the solution in the present paper to conserve energy, while the solution in Cramer and Mead 2020 [3] did not.
4. In the present paper, we explicitly check that our solution gives physically plausible photon transition times, even over astronomical distances between Emitter and Absorber Atoms, while the solution in Cramer and Mead 2020 [3] did not.

The present paper proposes a transfer of energy across space and time, with A being the degrees of freedom carrying the energy. In particular, this paper proposes that the vector potential A(t) (retarded or advanced) exists where no current J(r,t) is present. We make the assumption that A(t) is substantive everywhere, even when J(r,t)=0, and thus we may propose that the energy density P(x,t) is proportional to the product of the Emitter and Absorber vector potentials $A_{{⊥}_{E}}\left(x,t\right)\cdot A_{{⊥}_{A}}\left(x,t\right)$, as in Equation (33). This expression provides a much cleaner approach to visualizing the structure of the quantized field.

When Feynman and Dirac thought in terms of advanced and retarded potentials, they were not bothered by the question of whether the energy travels through space because they made an action-at-a-distance assumption. Both were trying to get rid of the infinities of classical electromagnetism theory, and they both failed to make the approach quantum. However, neither of them thought this approach applied to the bending of light by a mass. If they did, then they, too, would have worried about the energy transfer issue.

Wheeler and Feynman published their Absorber Theory papers in 1945 [32] and 1949 [33]. We know from Feynman’s Nobel Lecture that he abandoned this approach [34]. To our knowledge, Dirac never came back to it after his paper in 1936 [35]. Of course, soon after World War II, they would both have known that the Lamb and magnetic moment experiments appear to force one to come to grips with the radiation degrees of freedom in free space. To our knowledge, the last time that Feynman commented on action-at-a-distance with retarded and advanced potentials was the letter from Feynman to Wheeler in 1951 after the discovery of positronium [36]. It appears that Wheeler did not abandon direct-action theories, based on his favorable comments in Wesley and Wheeler 2003 [37].

In this paper, we have developed a mathematical/physical model of the complete energy transfer from an Emitter Atom to an Absorber Atom, compatible with the Transactional Interpretation of Quantum Mechanics, which exhibits the particle-like properties of traveling at the speed of light in a straight line from Emitter Atom to Absorber Atom in a vacuum in the absence of gravity, with the wave-like characteristics of an explicit physical wavelength, amplitude, frequency, velocity, and localized energy density, as illustrated in Figure 8. This new type of quantum object is neither a classical wave nor a classical particle, and we note that there is no existing name for a model of such a quantum object. So, we propose to call it a Wave–Particle Model, defined as the product of a retarded Emitter vector potential wave and an advanced Absorber vector potential wave, which exhibits the particle-like properties of losslessly carrying energy at the speed of light in a straight line from Emitter Atom to Absorber Atom in a vacuum in the absence of gravity.

We note also that our Wave–Particle Model gives a mathematical/physical description of the mysterious wave–particle duality phenomenon first described by de Broglie in 1924, but has never been mathematically modeled, as far as we know.

We note also that we have not demonstrated the more advanced test cases that we mentioned in the Introduction, namely:

- The single-slit experiment;
- The double-slit experiment;
- The double-slit experiment with detection at the slits;
- The Einstein–Podolsky–Rosen paradox;
- The Hanbury-Brown–Twiss effect;
- The Freedman–Clauser entanglement experiment.

These more advanced demonstrations will have to be the subject of future papers. But our Wave–Particle Model has been explicitly developed with both the wave-like and particle-like properties of photons in mind, and with the explicit inclusion of the Absorber Atom built in to the model as in TIQM, so it has been designed with all six test cases in mind, whereas QED does not provide a physical model that has been demonstrated to succeed on any of them, as far as we know.

Finally, we note that our present Wave–Particle Model does not address the other major problems and limitations of QED, listed in the Introduction, namely:

1. QED is not self-consistent.
2. Renormalization is not mathematically self-consistent.
4a. QED does not provide a corrected wavefunction model of an electron in a Hydrogen Atom, consistent with fine structure, spin ½ and Lamb Shift.
6. The Cosmological Constant Problem is still unresolved.
7. Standard QED cannot overcome Haag’s Inconsistency Theorem.

Resolution of these points will require work outside the scope of the current paper.

## Figures and Tables

**Table 1:** Model Parameters for various Emitter–Absorber distances.

**Arm’s Length**	**Earth to Sun**
d=1 m	$d=1.5\times 10^{11}\;m$
Travel time = $3.33564\times 10^{-9}\;s$	Travel time = 500.346 s
$\tau =5.57\times 10^{-16}\;s$	$\tau =2.16\times 10^{-10}\;s$
$c\tau =1.67\times 10^{-7}\;m$	cτ=0.0648 m
k=ωτ=8.6	$k=\omega \tau =3.348\times 10^{6}$
**One Light Year**	**HD 92740**
$d=9.461\times 10^{15}\;m$	$d=7.91\times 10^{19}\;m$
Travel time = $3.156\times 10^{7}\;s$	Travel time = $2.64\times 10^{11}\;s$
$\tau =5.43\times 10^{-8}\;s$	$\tau =4.96\times 10^{-6}\;s$
cτ=16.268 m	$c\tau =1.488\times 10^{3}\;m$
$k=\omega \tau =8.41\times 10^{8}$	$k=\omega \tau =7.69\times 10^{10}$
**Andromeda Galaxy**	**JADES-GS-z13-0**
$d=2.37\times 10^{22}\;m$	$d=1.2867\times 10^{26}\;m$
Travel time = $7.89\times 10^{13}\;s$	Travel time = $4.29\times 10^{17}\;s$
$\tau =8.58\times 10^{-5}\;s$	$\tau =6.328\times 10^{-3}\;s$
$c\tau =2.57\times 10^{4}\;m$	$c\tau =1.897\times 10^{6}\;m$
$k=\omega \tau =1.33\times 10^{12}$	$k=\omega \tau =9.81\times 10^{13}$

**Table 2:** Standard solutions of the Schrodinger Equation for the n = 1 state and n = 2 state.

**State**	**Normalized Radial Solution**	**Normalized Wavefunction Solution**
1S	$R_{1S}\left(r\right)=\frac{2}{{a_{0}}^{3/2}}e^{-\frac{r}{a_{0}}}$	$\psi_{1S}\left(r\right)=\frac{1}{\sqrt{\pi }{a_{0}}^{3/2}}e^{-\frac{r}{a_{0}}}$
2S	$R_{2S}\left(r\right)=\frac{1}{2\sqrt{2}{a_{0}}^{3/2}}\left(2-\frac{r}{a_{0}}\right)e^{-\frac{r}{2a_{0}}}$	$\psi_{2S}\left(r\right)=\frac{1}{4\sqrt{2\pi }{a_{0}}^{3/2}}\left(2-\frac{r}{a_{0}}\right)e^{-\frac{r}{2a_{0}}}$
2P	$R_{2P}\left(r\right)=\frac{1}{2\sqrt{6}{a_{0}}^{3/2}}\frac{r}{a_{0}}e^{-\frac{r}{2a_{0}}}$	$\psi_{2P}\left(r\right)=\frac{1}{4\sqrt{2\pi }{a_{0}}^{3/2}}\frac{r\cos\theta }{a_{0}}e^{-\frac{r}{2a_{0}}}$

**Figure A1:**
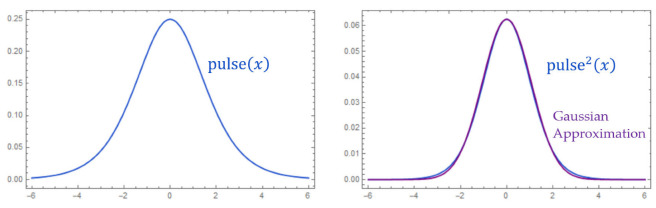
Pulse(x) and ${pulse}^{2}\left(x\right)$ functions, and the Gaussian approximation to ${pulse}^{2}\left(x\right)$.

**Figure A2:**
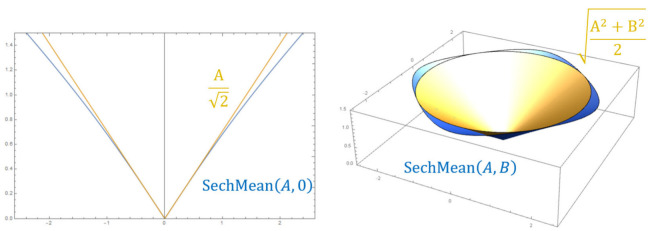
SechMean(A,B) and its canonical approximation.

## Data Availability

No new experimental data was collected in this research.

## Appendix A. Calculation of the Photon Energy Density

From Equation (38), we have

(A1) $$E\left(t\right)=C\frac{{\mu_{0}}^{2}\omega^{4}{D_{0}}^{2}}{16\pi^{2}}\int \sqrt{\frac{2}{T}\int_{t}^{t+T/2}{\left(\frac{sin\left(\theta \right)cos\left(\omega t_{E}\right)sin\left(\theta_{A}\right)cos\left(\omega t_{A}+\varphi \right)\;\hat{\theta }\cdot {\hat{\theta }}_{A}}{r\;\left(e^{t_{E}/\tau }+1\right)\left(e^{-t_{E}/\tau }+1\right)r_{A}\;\left(e^{t_{A}/\tau }+1\right)\left(e^{-t_{A}/\tau }+1\right)}\right)}^{2}\;dt}\;dvol$$

(A2) $$Units:\;\;\frac{q\;{sec}^{2}}{m\;V}\;{\left(\frac{V\;{sec}^{2}}{q\;m}\right)}^{2}\frac{1}{{sec}^{4}}{\left(\frac{q\;m}{sec}\right)}^{2}\;\frac{m^{3}}{m^{2}}=q\;V$$

We have found through numerical integrations that the terms sin(θ), $sin\left(\theta_{A}\right)$, and $\hat{\theta }\cdot {\hat{\theta }}_{A}$ can be neglected:

(A3) $$E\left(t\right)=C\frac{{\mu_{0}}^{2}\omega^{4}{D_{0}}^{2}}{16\pi^{2}}\int \sqrt{\frac{2}{T}\int_{t}^{t+T/2}{\left(\frac{cos\left(\omega t_{E}\right)cos\left(\omega t_{A}+\varphi \right)}{r\;\left(e^{t_{E}/\tau }+1\right)\left(e^{-t_{E}/\tau }+1\right)r_{A}\;\left(e^{t_{A}/\tau }+1\right)\left(e^{-t_{A}/\tau }+1\right)}\right)}^{2}\;dt}\;dvol$$

We can isolate the fine RMS temporal integration operations:

(A4) $$E\left(t\right)=C\frac{{\mu_{0}}^{2}\omega^{4}{D_{0}}^{2}}{16\pi^{2}}\int \frac{\sqrt{\frac{2}{T}\int_{t}^{t+T/2}{\left(cos\left(\omega t_{E}\right)cos\left(\omega t_{A}+\varphi \right)\right)}^{2}\;dt}}{r\;\left(e^{t_{E}/\tau }+1\right)\left(e^{-t_{E}/\tau }+1\right)r_{A}\;\left(e^{t_{A}/\tau }+1\right)\left(e^{-t_{A}/\tau }+1\right)}dvol$$

(A5) $$E\left(t\right)=C\frac{{\mu_{0}}^{2}\omega^{4}{D_{0}}^{2}}{16\pi^{2}}\int \frac{\sqrt{{\frac{2}{T}I}_{S}}}{r\;\left(e^{t_{E}/\tau }+1\right)\left(e^{-t_{E}/\tau }+1\right)r_{A}\;\left(e^{t_{A}/\tau }+1\right)\left(e^{-t_{A}/\tau }+1\right)}dvol$$

And now we must compute the square integral ${\;I}_{S}$:

(A6) $$I_{S}=\int_{t}^{t+T/2}{\left(cos\left(\omega t_{E}\right)cos\left(\omega t_{A}+\varphi \right)\right)}^{2}\;dt$$

(A7) $$=\int_{t}^{t+T/2}{cos}^{2}\left(\omega \left(t-\frac{r}{c}\right)\right){cos}^{2}\left(\omega \left(t+\frac{r_{A}}{c}\right)+\varphi \right)dt$$

(A8) $$=\int_{t}^{t+T/2}{cos}^{2}\left(\omega t-kr\right){cos}^{2}\left(\omega t+kr_{A}+\varphi \right)dt$$

where the wavenumber $k=\frac{\omega }{c}$, and the period $T=\frac{2\pi }{\omega }$.

(A9) $$\;\;\;=\int_{t}^{t+T/2}{\left(cos\left(\omega t\right)cos\left(kr\right)+sin\left(\omega t\right)sin\left(kr\right)\right)}^{2}{\left(cos\left(\omega t\right)cos\left(kr_{A}+\varphi \right)\;-sin\left(\omega t\right)sin\left(kr_{A}+\varphi \right)\right)}^{2}dt$$

(A10) $$\;\;\;=\int_{t}^{t+T/2}{\left({cos}^{2}\left(\omega t\right)cos\left(kr\right)cos\left(kr_{A}+\varphi \right)-cos\left(\omega t\right)sin\left(\omega t\right)cos\left(kr\right)sin\left(kr_{A}+\varphi \right)\;\;+cos\left(\omega t\right)sin\left(\omega t\right)sin\left(kr\right)cos\left(kr_{A}+\varphi \right)\;-{sin}^{2}\left(\omega t\right)sin\left(kr\right)sin\left(kr_{A}+\varphi \right)\right)}^{2}dt$$

(A11) $$=\int_{t}^{t+T/2}{\left({cos}^{2}\left(\omega t\right)cos\left(kr\right)cos\left(kr_{A}+\varphi \right)\;\;\;\;\;\;\;\;\;\;\;\;\;\;\;\;\;\;\;\;\;\;-cos\left(\omega t\right)sin\left(\omega t\right)\left(cos\left(kr\right)sin\left(kr_{A}+\varphi \right)-sin\left(kr\right)cos\left(kr_{A}+\varphi \right)\right)\;\;\;\;\;\;\;\;\;\;\;\;\;\;\;-\left(1-{cos}^{2}\left(\omega t\right)\right)sin\left(kr\right)sin\left(kr_{A}+\varphi \right)\right)}^{2}dt$$

(A12) $$=\int_{t}^{t+T/2}{\left({cos}^{2}\left(\omega t\right)\left(cos\left(kr\right)cos\left(kr_{A}+\varphi \right)+sin\left(kr\right)sin\left(kr_{A}+\varphi \right)\right)\;\;\;\;-cos\left(\omega t\right)sin\left(\omega t\right)\left(cos\left(kr\right)sin\left(kr_{A}+\varphi \right)\;\;\;\;\;\;\;\;-sin\left(kr\right)cos\left(kr_{A}+\varphi \right)\right)-sin\left(kr\right)sin\left(kr_{A}+\varphi \right)\right)}^{2}dt$$

(A13) $$\;\;\;=\int_{t}^{t+T/2}\left({cos}^{4}\left(\omega t\right){\left(cos\left(kr\right)cos\left(kr_{A}+\varphi \right)+sin\left(kr\right)sin\left(kr_{A}+\varphi \right)\right)}^{2}\;\;\;\;\;\;\;\;\;\;\;\;\;\;\;\;\;+{cos}^{2}\left(\omega t\right){sin}^{2}\left(\omega t\right){\left(cos\left(kr\right)sin\left(kr_{A}+\varphi \right)-sin\left(kr\right)cos\left(kr_{A}+\varphi \right)\right)}^{2}\;+{sin}^{2}\left(kr\right){sin}^{2}\left(kr_{A}+\varphi \right)\;\;\;\;\;\;\;\;-2{cos}^{3}\left(\omega t\right)sin\left(\omega t\right)\left(cos\left(kr\right)cos\left(kr_{A}+\varphi \right)\;\;\;\;\;\;\;\;\;\;\;\;\;\;\;\;\;\;+sin\left(kr\right)sin\left(kr_{A}+\varphi \right)\right)\left(cos\left(kr\right)sin\left(kr_{A}+\varphi \right)-sin\left(kr\right)cos\left(kr_{A}+\varphi \right)\right)\;\;\;\;\;\;-2{cos}^{2}\left(\omega t\right)\left(cos\left(kr\right)cos\left(kr_{A}+\varphi \right)\;\;\;\;\;\;\;\;\;\;+sin\left(kr\right)sin\left(kr_{A}+\varphi \right)\right)sin\left(kr\right)sin\left(kr_{A}+\varphi \right)\;\;\;\;\;\;\;\;+2cos\left(\omega t\right)sin\left(\omega t\right)\left(cos\left(kr\right)sin\left(kr_{A}+\varphi \right)\;\;\;\;\;\;\;\;\;\;\;-sin\left(kr\right)cos\left(kr_{A}+\varphi \right)\right)sin\left(kr\right)sin\left(kr_{A}+\varphi \right)\right)dt$$

(A14) $$\;\;\;\;\;\;={\left(cos\left(kr\right)cos\left(kr_{A}+\varphi \right)+sin\left(kr\right)sin\left(kr_{A}+\varphi \right)\right)}^{2}\int_{t}^{t+T/2}{cos}^{4}\left(\omega t\right)\;dt\;\;\;\;+{\left(cos\left(kr\right)sin\left(kr_{A}+\varphi \right)\;-sin\left(kr\right)cos(kr_{A}\;\;\;\;\;\;\;+\varphi )\right)}^{2}\int_{t}^{t+T/2}{cos}^{2}\left(\omega t\right){sin}^{2}\left(\omega t\right)\;dt\;\;\;\;\;\;\;\;+{sin}^{2}\left(kr\right){sin}^{2}\left(kr_{A}+\varphi \right)\int_{t}^{t+T/2}dt\;\;\;\;-2\left(cos\left(kr\right)cos\left(kr_{A}+\varphi \right)\;\;\;\;\;\;\;\;\;\;\;\;+sin\left(kr\right)sin\left(kr_{A}+\varphi \right)\right)\left(cos\left(kr\right)sin\left(kr_{A}+\varphi \right)\;\;\;\;\;\;\;\;\;\;\;\;\;-sin\left(kr\right)cos\left(kr_{A}+\varphi \right)\right)\int_{t}^{t+\frac{T}{2}}{cos}^{3}\left(\omega t\right)sin\left(\omega t\right)\;dt\;\;\;\;\;\;\;\;\;\;\;\;\;\;\;\;\;\;\;-2\left(cos\left(kr\right)cos\left(kr_{A}+\varphi \right)+sin\left(kr\right)sin\left(kr_{A}+\varphi \right)\right)sin\left(kr\right)sin(kr_{A}\;\;\;+\varphi )\int_{t}^{t+T/2}{cos}^{2}\left(\omega t\right)\;dt\;\;\;\;\;\;\;\;\;\;\;\;\;\;\;\;\;\;\;+2\left(cos\left(kr\right)sin\left(kr_{A}+\varphi \right)-sin\left(kr\right)cos\left(kr_{A}+\varphi \right)\right)sin\left(kr\right)sin(kr_{A}\;\;\;\;\;\;+\varphi )\int_{t}^{t+T/2}cos\left(\omega t\right)sin\left(\omega t\right)\;dt\;$$

(A15) $$\;\;\;\;\;={\left(cos\left(kr\right)cos\left(kr_{A}+\varphi \right)+sin\left(kr\right)sin\left(kr_{A}+\varphi \right)\right)}^{2}\left(\frac{3\pi }{8\omega }\right)\;\;\;\;\;\;\;+{\left(cos\left(kr\right)sin\left(kr_{A}+\varphi \right)\;\;\;\;\;\;\;\;\;\;-sin\left(kr\right)cos\left(kr_{A}+\varphi \right)\right)}^{2}\left(\frac{\pi }{8\omega }\right)\;\;\;\;\;\;\;\;\;\;+{sin}^{2}\left(kr\right){sin}^{2}\left(kr_{A}+\varphi \right)\left(\frac{\pi }{\omega }\right)\;\;\;\;\;\;\;\;-2\left(cos\left(kr\right)cos\left(kr_{A}+\varphi \right)\;\;\;\;\;\;\;\;\;\;\;\;\;\;\;\;\;+sin\left(kr\right)sin\left(kr_{A}+\varphi \right)\right)\left(cos\left(kr\right)sin\left(kr_{A}+\varphi \right)\;\;\;\;\;\;\;\;\;\;-sin\left(kr\right)cos\left(kr_{A}+\varphi \right)\right)\left(0\right)\;\;\;\;\;\;\;\;\;\;\;\;\;\;\;\;\;\;\;\;\;\;-2\left(cos\left(kr\right)cos\left(kr_{A}+\varphi \right)+sin\left(kr\right)sin\left(kr_{A}+\varphi \right)\right)sin\left(kr\right)sin(kr_{A}\;\;+\varphi )\left(\frac{\pi }{2\omega }\right)\;\;\;\;\;\;\;\;\;\;\;\;\;\;\;\;\;\;\;\;\;\;+2\left(cos\left(kr\right)sin\left(kr_{A}+\varphi \right)-sin\left(kr\right)cos\left(kr_{A}+\varphi \right)\right)sin\left(kr\right)sin(kr_{A}\;+\varphi )\left(0\right)$$

(A16) $$\;\;\;\;={\left(cos\left(kr\right)cos\left(kr_{A}+\varphi \right)+sin\left(kr\right)sin\left(kr_{A}+\varphi \right)\right)}^{2}\left(\frac{3\pi }{8\omega }\right)\;\;\;\;\;\;\;\;+{\left(cos\left(kr\right)sin\left(kr_{A}+\varphi \right)\;\;\;\;\;\;\;\;\;\;-sin\left(kr\right)cos\left(kr_{A}+\varphi \right)\right)}^{2}\left(\frac{\pi }{8\omega }\right)\;\;\;\;\;\;\;\;\;\;+{sin}^{2}\left(kr\right){sin}^{2}\left(kr_{A}+\varphi \right)\left(\frac{\pi }{\omega }\right)\;\;\;\;\;\;\;\;\;\;\;\;\;\;\;\;\;\;\;\;\;-2\left(cos\left(kr\right)cos\left(kr_{A}+\varphi \right)+sin\left(kr\right)sin\left(kr_{A}+\varphi \right)\right)sin\left(kr\right)sin(kr_{A}\;+\varphi )\left(\frac{\pi }{2\omega }\right)$$

(A17) $$\;\;\;\;={\left(cos\left(kr\right)cos\left(kr_{A}+\varphi \right)+sin\left(kr\right)sin\left(kr_{A}+\varphi \right)\right)}^{2}\left(\frac{3\pi }{8\omega }\right)\;\;\;\;\;\;+{\left(cos\left(kr\right)sin\left(kr_{A}+\varphi \right)\;\;\;\;\;\;\;\;\;-sin\left(kr\right)cos\left(kr_{A}+\varphi \right)\right)}^{2}\left(\frac{\pi }{8\omega }\right)\;\;\;\;\;\;\;\;+{sin}^{2}\left(kr\right){sin}^{2}\left(kr_{A}+\varphi \right)\left(\frac{\pi }{\omega }\right)\;\;\;\;\;\;\;\;\;\;\;\;\;\;\;\;\;\;\;\;\;-\left(cos\left(kr\right)cos\left(kr_{A}+\varphi \right)+sin\left(kr\right)sin\left(kr_{A}+\varphi \right)\right)sin\left(kr\right)sin(kr_{A}\;+\varphi )\left(\frac{\pi }{\omega }\right)$$

(A18) $$={\left(cos\left(kr\right)cos\left(kr_{A}+\varphi \right)+sin\left(kr\right)sin\left(kr_{A}+\varphi \right)\right)}^{2}\left(\frac{3\pi }{8\omega }\right)\;\;\;+{\left(cos\left(kr\right)sin\left(kr_{A}+\varphi \right)\;\;\;\;\;\;-sin\left(kr\right)cos\left(kr_{A}+\varphi \right)\right)}^{2}\left(\frac{\pi }{8\omega }\right)\;\;\;\;\;\;\;\;\;\;\;\;\;-\left(cos\left(kr\right)cos\left(kr_{A}+\varphi \right)\right)sin\left(kr\right)sin\left(kr_{A}+\varphi \right)\left(\frac{\pi }{\omega }\right)$$

(A19) $$={\left(cos\left(kr-kr_{A}-\varphi \right)\right)}^{2}\left(\frac{3\pi }{8\omega }\right)\;+{\left(sin\left(kr-kr_{A}-\varphi \right)\right)}^{2}\left(\frac{\pi }{8\omega }\right)\;\;\;\;\;\;\;\;\;-cos\left(kr\right)cos\left(kr_{A}+\varphi \right)sin\left(kr\right)sin\left(kr_{A}+\varphi \right)\left(\frac{\pi }{\omega }\right)$$

(A20) $$={\left(cos\left(kr-kr_{A}-\varphi \right)\right)}^{2}\left(\frac{2\pi }{8\omega }\right)+{\left(cos\left(kr-kr_{A}-\varphi \right)\right)}^{2}\left(\frac{\pi }{8\omega }\right)\;\;+{\left(sin\left(kr-kr_{A}-\varphi \right)\right)}^{2}\left(\frac{\pi }{8\omega }\right)\;\;\;\;\;\;\;\;\;-cos\left(kr\right)cos\left(kr_{A}+\varphi \right)sin\left(kr\right)sin\left(kr_{A}+\varphi \right)\left(\frac{\pi }{\omega }\right)$$

(A21) $$=\frac{\pi }{8\omega }+{\left(cos\left(kr\right)cos\left(kr_{A}+\varphi \right)+sin\left(kr\right)sin\left(kr_{A}+\varphi \right)\right)}^{2}\left(\frac{\pi }{4\omega }\right)\;\;\;\;\;\;\;\;\;\;-cos\left(kr\right)cos\left(kr_{A}+\varphi \right)sin\left(kr\right)sin\left(kr_{A}+\varphi \right)\left(\frac{\pi }{\omega }\right)$$

(A22) $$\;\;\;\;=\frac{\pi }{8\omega }+\left({cos}^{2}\left(kr\right){cos}^{2}\left(kr_{A}+\varphi \right)+2cos\left(kr\right)cos\left(kr_{A}+\varphi \right)sin\left(kr\right)sin\left(kr_{A}+\varphi \right)\;+{sin}^{2}\left(kr\right){sin}^{2}\left(kr_{A}+\varphi \right)\right)\left(\frac{\pi }{4\omega }\right)\;\;\;\;\;\;\;\;-cos\left(kr\right)cos\left(kr_{A}+\varphi \right)sin\left(kr\right)sin\left(kr_{A}+\varphi \right)\left(\frac{\pi }{\omega }\right)$$

(A23) $$\;\;\;\;=\frac{\pi }{8\omega }+\left({cos}^{2}\left(kr\right){cos}^{2}\left(kr_{A}+\varphi \right)-2cos\left(kr\right)cos\left(kr_{A}+\varphi \right)sin\left(kr\right)sin\left(kr_{A}+\varphi \right)\;+{sin}^{2}\left(kr\right){sin}^{2}\left(kr_{A}+\varphi \right)\right)\left(\frac{\pi }{4\omega }\right)\;\;\;\;$$

(A24) $$=\frac{\pi }{8\omega }+{\left(cos\left(kr\right)cos\left(kr_{A}+\varphi \right)-sin\left(kr\right)sin\left(kr_{A}+\varphi \right)\right)}^{2}\left(\frac{\pi }{4\omega }\right)$$

(A25) $$=\frac{\pi }{8\omega }+{\left(cos\left(kr+kr_{A}+\varphi \right)\right)}^{2}\left(\frac{\pi }{4\omega }\right)$$

(A26) $$=\frac{\pi }{8\omega }+{\left(cos\left(k\left(r+r_{A}\right)+\varphi \right)\right)}^{2}\left(\frac{\pi }{4\omega }\right)$$

(A27) $$=\frac{\pi }{8\omega }\left(1+2{cos}^{2}\left(k\left(r+r_{A}\right)+\varphi \right)\right)$$

(A28) $$=\frac{\pi }{8\omega }\left(1+1+cos\left(2k\left(r+r_{A}\right)+2\varphi \right)\right)$$

(A29) $$I_{S}=\frac{\pi }{8\omega }\left(2+cos\left(2k\left(r+r_{A}\right)+2\varphi \right)\right)$$

(A30) $$I_{S}=\frac{\pi }{4\omega }\left(1+\frac{1}{2}cos\left(2k\left(r+r_{A}\right)+2\varphi \right)\right)$$

(A31) $$E\left(t\right)=C\frac{{\mu_{0}}^{2}\omega^{4}{D_{0}}^{2}}{16\pi^{2}}\int \frac{\sqrt{\;\frac{1}{8}\left(2+cos\left(2k\left(r+r_{A}\right)+2\varphi \right)\right)}}{r\;\left(e^{t_{E}/\tau }+1\right)\left(e^{-t_{E}/\tau }+1\right)r_{A}\;\left(e^{t_{A}/\tau }+1\right)\left(e^{-t_{A}/\tau }+1\right)}dvol$$

(A32) $$E\left(t\right)=C\frac{{\mu_{0}}^{2}\omega^{4}{D_{0}}^{2}}{32\pi^{2}}\int \frac{\sqrt{\;\frac{1}{2}\left(2+cos\left(2k\left(r+r_{A}\right)+2\varphi \right)\right)}}{r\;\left(e^{t_{E}/\tau }+1\right)\left(e^{-t_{E}/\tau }+1\right)r_{A}\;\left(e^{t_{A}/\tau }+1\right)\left(e^{-t_{A}/\tau }+1\right)}dvol$$

(A33) $$E\left(t\right)=C\frac{{\mu_{0}}^{2}\omega^{4}{D_{0}}^{2}}{32\pi^{2}}\int \frac{\sqrt{\;1+\frac{1}{2}cos\left(2k\left(r+r_{A}\right)+2\varphi \right)}}{r\;\left(e^{t_{E}/\tau }+1\right)\left(e^{-t_{E}/\tau }+1\right)r_{A}\;\left(e^{t_{A}/\tau }+1\right)\left(e^{-t_{A}/\tau }+1\right)}dvol$$

## Appendix B. Analytic Integration of the Total Energy in Transit

We now proceed with the analytic integration of Equation (42):

(A34) $$E\left(\xi \right)=C\frac{{\mu_{0}}^{2}\omega^{4}{D_{0}}^{2}}{32\pi^{2}}\int \frac{\sqrt{\;1+\frac{1}{2}cos\left(\frac{2k}{c\tau }\left(r_{E}+r_{A}\right)\right)}}{r_{E}\;r_{A}\;\left(e^{\frac{r_{EE}}{c\tau }}+1\right)\left(e^{-\frac{r_{EE}}{c\tau }}+1\right)\left(e^{\frac{r_{AA}}{c\tau }}+1\right)\left(e^{-\frac{r_{AA}}{c\tau }}+1\right)}\;dvol$$

We can rewrite Equation (A34) as

(A35) $$E\left(\xi \right)=C\frac{{\mu_{0}}^{2}\omega^{4}{D_{0}}^{2}}{32\pi^{2}}\int \frac{\sqrt{\;1+\frac{1}{2}cos\left(\frac{2k}{c\tau }\left(r_{E}+r_{A}\right)\right)}pulse\left(\frac{r_{EE}}{c\tau }\right)pulse\left(\frac{r_{AA}}{c\tau }\right)}{r_{E}\;r_{A}\;}\;dvol$$

where we have defined the pulse(x) function as

(A36) $$pulse\left(x\right)=\frac{1}{\left(e^{x}+1\right)\left(e^{-x}+1\right)}=\frac{{sech}^{2}\left(x/2\right)}{4}$$

(A37) $${pulse}^{2}\left(x\right)=\frac{1}{{\left(e^{x}+1\right)}^{2}{\left(e^{-x}+1\right)}^{2}}=\frac{{sech}^{4}\left(x/2\right)}{16}\approx \frac{1}{16}{\;e}^{-\frac{{9\pi x}^{2}}{64}}$$

where the Gaussian coefficient $\frac{9\pi }{64}$ was chosen so that the integrals of the exact function and the approximation are equal:

(A38) $$\int_{-\infty }^{\infty }\frac{1}{16}{\;e}^{-a\;x^{2}}dx=\frac{1}{16}\sqrt{\frac{\pi }{a}}=\int_{-\infty }^{\infty }\frac{1}{{\left(e^{x}+1\right)}^{2}{\left(e^{-x}+1\right)}^{2}}dx=\frac{1}{6}\;\;\;\;\;\;\;\;\;\;\Rightarrow \;\;\;\;\;\;\;\;a=\frac{9\pi }{64}$$

The pulse(x) and ${pulse}^{2}\left(x\right)$ functions are shown in Figure A1, along with the Gaussian approximation to the ${pulse}^{2}\left(x\right)$ function.

Equation (A35) contains the product of two spherical pulse(x) functions: $pulse\left(\frac{r_{EE}}{c\tau }\right)pulse\left(\frac{r_{AA}}{c\tau }\right)$. We will convert this into a new ${pulse}^{2}\left(x\right)$ function of the intersection function $f\left(\frac{r_{EE}}{c\tau },\frac{r_{AA}}{c\tau }\right)$.

(A39) $${pulse}^{2}\left(f\left(\frac{r_{EE}}{c\tau },\frac{r_{AA}}{c\tau }\right)\right)=pulse\left(\frac{r_{EE}}{c\tau }\right)pulse\left(\frac{r_{AA}}{c\tau }\right)$$

(A40) $$pulse\left(f\left(\frac{r_{EE}}{c\tau },\frac{r_{AA}}{c\tau }\right)\right)=\sqrt{pulse\left(\frac{r_{EE}}{c\tau }\right)pulse\left(\frac{r_{AA}}{c\tau }\right)}$$

(A41) $$f\left(\frac{r_{EE}}{c\tau },\frac{r_{AA}}{c\tau }\right)={pulse}^{-1}\left(\sqrt{pulse\left(\frac{r_{EE}}{c\tau }\right)pulse\left(\frac{r_{AA}}{c\tau }\right)}\right)$$

(A42) $$f\left(\frac{r_{EE}}{c\tau },\frac{r_{AA}}{c\tau }\right)={pulse}^{-1}\left(\frac{1}{4}sech\left(\frac{r_{EE}}{2c\tau }\right)sech\left(\frac{r_{AA}}{2c\tau }\right)\right)$$

We can derive from Equation (A36) that

(A43) $${pulse}^{-1}\left(g\right)=x={2sech}^{-1}\left(2\sqrt{g}\right)\;\;\;\;\;\;\;\;\;\;\;\;\;0\leq f\leq 1/4$$

Therefore,

(A44) $$f\left(\frac{r_{EE}}{c\tau },\frac{r_{AA}}{c\tau }\right)={2sech}^{-1}\left(\sqrt{sech\left(\frac{r_{EE}}{2c\tau }\right)sech\left(\frac{r_{AA}}{2c\tau }\right)}\right)$$

Let us define a new function

(A45) $$SechMean\left(A,B\right)={sech}^{-1}\left(\sqrt{sech\left(A\right)sech\left(B\right)}\right)$$

So then,

(A46) $$f\left(\frac{r_{EE}}{c\tau },\frac{r_{AA}}{c\tau }\right)=2\;SechMean\left(\frac{r_{EE}}{2c\tau },\frac{r_{AA}}{2c\tau }\right)$$

And then we have

(A47) $$E\left(\xi \right)=C\frac{{\mu_{0}}^{2}\omega^{4}{D_{0}}^{2}}{32\pi^{2}}\int \frac{\sqrt{\;1+\frac{1}{2}cos\left(\frac{2k}{c\tau }\left(r_{E}+r_{A}\right)\right)}{pulse}^{2}\left(2\;SechMean\left(\frac{r_{EE}}{2c\tau },\frac{r_{AA}}{2c\tau }\right)\right)}{r_{E}\;r_{A}\;}\;dvol$$

This exact function cannot be integrated in closed form. Now, we will make our first approximation:

(A48) $$SechMean\left(A,B\right)\;\approx \sqrt{\frac{A^{2}+B^{2}}{2}}$$

Substituting into Equation (A34) gives

(A49) $$E\left(\xi \right)\approx C\frac{{\mu_{0}}^{2}\omega^{4}{D_{0}}^{2}}{32\pi^{2}}\int \frac{\sqrt{\;1+\frac{1}{2}cos\left(\frac{2k}{c\tau }\left(r_{E}+r_{A}\right)\right)}{pulse}^{2}\left(\sqrt{2\left({\left(\frac{r_{EE}}{2c\tau }\right)}^{2}+{\left(\frac{r_{AA}}{2c\tau }\right)}^{2}\right)}\right)}{r_{E}\;r_{A}\;}\;dvol$$

And then using the Gaussian approximation for ${pulse}^{2}\left(x\right)$ from Equation (A37) gives

(A50) $$E\left(\xi \right)\approx C\frac{{\mu_{0}}^{2}\omega^{4}{D_{0}}^{2}}{32\pi^{2}}\frac{1}{16}\int \frac{\sqrt{\;1+\frac{1}{2}cos\left(\frac{2k}{c\tau }\left(r_{E}+r_{A}\right)\right)}{\;e}^{-\frac{9\pi \left({r_{EE}}^{2}+{r_{AA}}^{2}\right)}{32{\left(c\tau \right)}^{2}}}\;}{r_{E}\;r_{A}\;}\;dvol$$

Now we will shift the coordinate system origin to the center of the photon with x⟹x+ξd:

(A51) $$r_{E}=\sqrt{{\left(x+\xi d\right)}^{2}+y^{2}+z^{2}}\;\;\;\;\;\;\;\;\;\;\;\;\;\;\;\;r_{A}=\sqrt{{\left(d-\left(x+\xi d\right)\right)}^{2}+y^{2}+z^{2}}$$

(A52) $$r_{EE}=r_{E}-\xi d\;\;\;\;\;\;\;\;\;\;\;\;\;\;\;\;r_{AA}=r_{A}-\left(1-\xi \right)d$$

And use the Taylor Series approximation

(A53) $$\sqrt{1+x}\approx 1+\frac{x}{2}-\frac{x^{2}}{8}+\ldots$$

The integral at the halfway point $\xi =\frac{1}{2}$ is

(A54) $$E(\frac{1}{2})\approx C\frac{{\mu_{0}}^{2}\omega^{4}{D_{0}}^{2}}{32\pi^{2}}\frac{1}{4d^{2}}\int \sqrt{\;1+\frac{1}{2}cos\left(\frac{2k}{c\tau }\left(\frac{3x^{2}+{4y}^{2}+4z^{2}}{2d}\right)\right)}e^{-\frac{9\pi \left(\frac{y^{4}+z^{4}}{d^{2}}+x^{2}\right)}{64{\left(c\tau \right)}^{2}}}\;dvol$$

And the integral can be done in closed form with the second-order Taylor series approximation for the square root function

(A55) $$E(\frac{1}{2})\approx C\frac{{\mu_{0}}^{2}\omega^{4}{D_{0}}^{2}}{32\pi^{2}}\frac{1}{4d^{2}}\int (1+\frac{1}{4}cos\left(\frac{2k}{c\tau }\left(\frac{3x^{2}+{4y}^{2}+4z^{2}}{2d}\right)\right)\;\;\;\;\;\;\;\;\;\;\;\;\;\;-\frac{1}{32}{cos}^{2}\left(\frac{2k}{c\tau }\left(\frac{3x^{2}+{4y}^{2}+4z^{2}}{2d}\right)\right))e^{-\frac{9\pi \left(\frac{y^{4}+z^{4}}{d^{2}}+x^{2}\right)}{64{\left(c\tau \right)}^{2}}}\;dvol$$

leading to

(A56) $$E(\frac{1}{2})\approx C\frac{{\mu_{0}}^{2}\omega^{4}{D_{0}}^{2}}{32\pi^{2}}\left(-\frac{\Gamma \left(-\frac{3}{4}\right)\Gamma \left(\frac{1}{4}\right)}{3\sqrt{\pi }\;}\frac{{S\left(k\right)\left(c\tau \right)}^{2}}{d\;}\right)$$

where the Path Fraction S(k) is defined as

(A57) $$S\left(k\right)=\frac{\int \sqrt{1+\frac{1}{2}cos\left(\frac{2k}{c\tau }\left(\frac{3x^{2}+{4y}^{2}+4z^{2}}{2d}\right)\right)}e^{-\frac{9\pi \left(\frac{y^{4}+z^{4}}{d^{2}}+x^{2}\right)}{64{\left(c\tau \right)}^{2}}}\;dvol}{\int e^{-\frac{9\pi \left(\frac{y^{4}+z^{4}}{d^{2}}+x^{2}\right)}{64{\left(c\tau \right)}^{2}}}\;dvol}$$

We find

(A58) $$S\left(k\right)\approx \frac{63}{64}$$

We can see this directly by using

(A59) $${cos}^{2}\left(x\right)=\frac{1+cos\left(2x\right)}{2}$$

In Equation (A55), we have

(A60) $$E\left(\frac{1}{2}\right)\approx C\frac{{\mu_{0}}^{2}\omega^{4}{D_{0}}^{2}}{32\pi^{2}}\frac{1}{4d^{2}}\int \left(1+\frac{1}{4}cos\left(\frac{2k}{c\tau }\left(\frac{3x^{2}+{4y}^{2}+4z^{2}}{2d}\right)\right)\;\;\;\;\;\;\;\;\;\;\;\;\;\;-\frac{1}{64}\left(1+cos\left(\frac{4k}{c\tau }\left(\frac{3x^{2}+{4y}^{2}+4z^{2}}{2d}\right)\right)\right)\right)e^{-\frac{9\pi \left(\frac{y^{4}+z^{4}}{d^{2}}+x^{2}\right)}{64{\left(c\tau \right)}^{2}}}\;dvol$$

(A61) $$E\left(\frac{1}{2}\right)\approx C\frac{{\mu_{0}}^{2}\omega^{4}{D_{0}}^{2}}{32\pi^{2}}\frac{1}{4d^{2}}\int \left(\frac{63}{64}+\frac{1}{4}cos\left(\frac{2k}{c\tau }\left(\frac{3x^{2}+{4y}^{2}+4z^{2}}{2d}\right)\right)\;\;\;\;\;\;\;\;\;\;\;\;\;\;\;-\frac{1}{64}\left(cos\left(\frac{4k}{c\tau }\left(\frac{3x^{2}+{4y}^{2}+4z^{2}}{2d}\right)\right)\right)\right)e^{-\frac{9\pi \left(\frac{y^{4}+z^{4}}{d^{2}}+x^{2}\right)}{64{\left(c\tau \right)}^{2}}}\;dvol$$
